# Ageing rate of older adults affects the factors associated with, and the determinants of malnutrition in the community: a systematic review and narrative synthesis

**DOI:** 10.1186/s12877-021-02583-2

**Published:** 2021-12-04

**Authors:** Laura A. Bardon, Clare A. Corish, Meabh Lane, Maria Gabriella Bizzaro, Katherine Loayza Villarroel, Michelle Clarke, Lauren C. Power, Eileen R. Gibney, Patricia Dominguez Castro

**Affiliations:** 1 School of Agriculture and Food Science, University College Dublin, Dublin, Republic of Ireland; 2 Institute of Food and Health, University College Dublin, Dublin, Republic of Ireland; 3 School of Public Health, Physiotherapy and Sports Science, University College Dublin, Dublin, Republic of Ireland

**Keywords:** Undernutrition, Older adults, Determinants, Malnutrition

## Abstract

**Background:**

Malnutrition negatively impacts on health, quality of life and disease outcomes in older adults. The reported factors associated with, and determinants of malnutrition, are inconsistent between studies. These factors may vary according to differences in rate of ageing. This review critically examines the evidence for the most frequently reported sociodemographic factors and determinants of malnutrition and identifies differences according to rates of ageing.

**Methods:**

A systematic search of the PubMed Central and Embase databases was conducted in April 2019 to identify papers on ageing and poor nutritional status. Numerous factors were identified, including factors from demographic, food intake, lifestyle, social, physical functioning, psychological and disease-related domains. Where possible, community-dwelling populations assessed within the included studies (*N* = 68) were categorised according to their ageing rate: ‘successful’, ‘usual’ or ‘accelerated’.

**Results:**

Low education level and unmarried status appear to be more frequently associated with malnutrition within the successful ageing category. Indicators of declining mobility and function are associated with malnutrition and increase in severity across the ageing categories. Falls and hospitalisation are associated with malnutrition irrespective of rate of ageing. Factors associated with malnutrition from the food intake, social and disease-related domains increase in severity in the accelerated ageing category. Having a cognitive impairment appears to be a determinant of malnutrition in successfully ageing populations whilst dementia is reported to be associated with malnutrition within usual and accelerated ageing populations.

**Conclusions:**

This review summarises the factors associated with malnutrition and malnutrition risk reported in community-dwelling older adults focusing on differences identified according to rate of ageing. As the rate of ageing speeds up, an increasing number of factors are reported within the food intake, social and disease-related domains; these factors increase in severity in the accelerated ageing category. Knowledge of the specific factors and determinants associated with malnutrition according to older adults’ ageing rate could contribute to the identification and prevention of malnutrition. As most studies included in this review were cross-sectional, longitudinal studies and meta-analyses comprehensively assessing potential contributory factors are required to establish the true determinants of malnutrition.

## Background

Improvements in healthcare, along with the development of medical treatments and vaccines, have increased life expectancy worldwide [1]. This has radically changed the global population demographic, with the proportion of older adults increasing, especially in developed countries [2]. Within Europe, 19.2% of the population were aged 65 years or over in 2016, with a projected increase to 29.1% by 2080 [3]. Considerable challenges arise with this increasing ageing population, among them promoting good health and well-being within this group so that they can live independently in the community for as long as possible [4].

One such challenge among older adults living in the community is risk of malnutrition and more specifically undernutrition (hereafter referred to as malnutrition) [5, 6]. Older adults are at increased risk of developing malnutrition due to natural age-related changes [7], namely, unfavourable changes in body composition, increased requirements for protein and certain micronutrients, alterations in appetite and declining sensory function. Left untreated, malnutrition can detrimentally affect cognitive and physical function, both of which can lead to loss of independence, increased risk of disease and poorer health outcomes [8–11]. Moreover, malnutrition is a complex multifactorial process, with many other components, such as, lifestyle, financial, social, psychological, presence of disease and medication use, known to contribute [12]. Within the published literature, there is little consistency between previously reported factors associated with malnutrition. In developed countries, malnutrition prevalence differs across community and healthcare settings depending on the individual’s characteristics, and the tools used to identify malnutrition. The greatest number of malnourished older adults in the UK is in the community setting (accounting for approximately 5% of the older population) [13, 14]. Community-dwelling older adults are a heterogeneous group who may experience remarkable differences in their ageing trajectory; namely, successful, usual or accelerated rates [15]. Successfully ageing older adults have few health conditions, are independent, rarely use healthcare services and their years of ill health are condensed into the end-of-life. Usually ageing older adults typically maintain their functional ability and independence but have health conditions and require frequent visits to their general practitioner (GP) to maintain their health status. Those experiencing an accelerated rate of ageing are frailer and more dependent than expected for their age, have multiple chronic diseases or experience rapid disease progression, and are frequent users of healthcare services [15].

With the global increase in life expectancy, more attention is being drawn to different rates of ageing. In particular, the concept of successful ageing is now acknowledged to be an important area of research. Nonetheless, whilst there is general agreement on the characteristics typical of a person ageing at a successful rate, to date, there is no consensus on how this concept should be defined. One of the most used definitions for successful ageing is someone who is ‘free of disease and disability, has a high physical and cognitive functioning ability and has an active engagement with life in general’ [16]. Rate of ageing can be influenced both positively and negatively by lifestyle, diet, psychological, psychosocial and disease related factors. Higher rates of physical activity throughout life are strongly linked to successful ageing [17, 18]. Older adults who self-report good health and no pain are more likely to age successfully than those that don’t [19]. Older adults experiencing different ageing trajectories may have different determinants of malnutrition which are specific to their rate of ageing.

Malnutrition in older adults is often under-recognised and poorly managed [20]. This can be attributed to the fact that it is a slow progressing condition and, therefore, its early signs and symptoms are not easily recognised either by affected individuals [21] or healthcare professionals (HCPs) [22]. Additionally, a universal definition and agreed diagnostic criteria have only recently emerged [20, 23, 24]. With the aim of achieving consensus on the definition of malnutrition, the European Society for Clinical Nutrition and Metabolism (ESPEN) stated (in 2015) that the following definition of malnutrition was generally accepted; “a state resulting from lack of uptake or intake of nutrition leading to altered body composition (decreased FFM) and body cell mass leading to diminished physical and mental function and impaired clinical outcome from disease” [25]. Furthermore, a global consensus for the diagnosis of malnutrition has recently (2019) been published based on a two-step approach; screening for risk of malnutrition using a validated tool, followed by assessment of the condition to provide a diagnosis of malnutrition and to grade its severity [26, 27].

Understanding and identifying factors that lead to malnutrition is critical for developing interventions aimed at preventing or delaying disability in older adults. This is particularly important in the community, where although prevalence is low, the greatest number of at-risk individuals reside [14, 28]. Community-dwelling older adults are a heterogeneous group; thus, the factors related to, or determinants of, malnutrition may vary according to individual differences in the rate of ageing. Potential differences in determinants of, and factors related to malnutrition according to differences in ageing rates may contribute to the heterogeneity between currently published studies. The aim of this review, therefore, is to summarise the current evidence relating to the sociodemographic factors associated with, and determinants of, malnutrition and malnutrition risk in community-dwelling older populations and, to explore potential differences according to different rates of ageing [15].

## Methods

### Search strategy

**Fig. 1:**
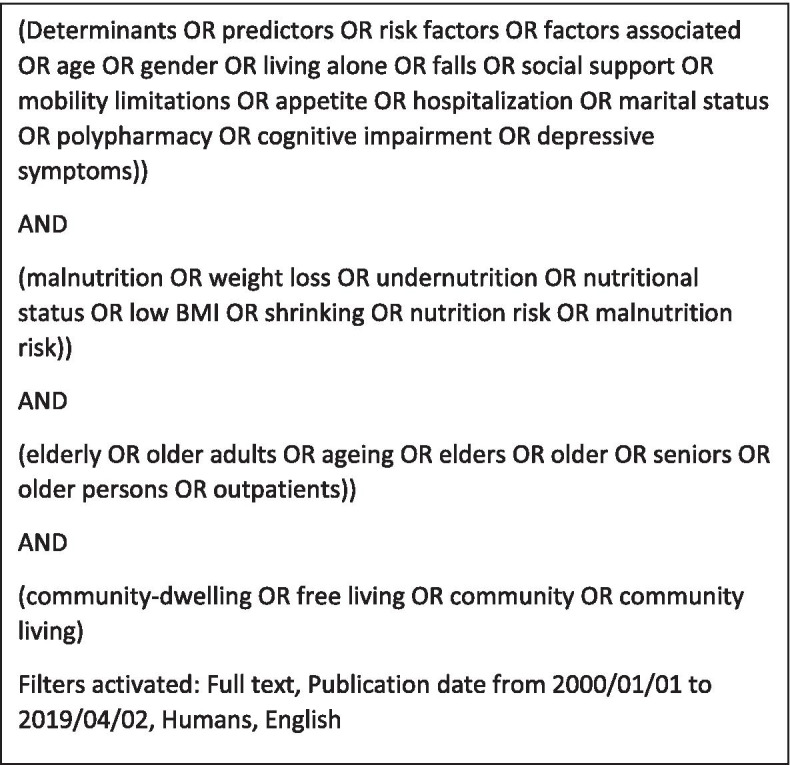
Search terms

Two independent systematic searches (Search 1, LAB; Search 2, KL, ML, MGB) of PubMed Central and Embase databases were conducted in April 2019 to identify relevant papers on ageing and poor nutritional status. Duplicates were excluded (LAB and KL), and titles examined to assess suitability for inclusion (LAB and ML). Studies examining the sociodemographic factors associated with, or determinants of, malnutrition were included. The key search terms were as follows: the primary outcome (protein-energy malnutrition, malnutrition, undernutrition, weight loss, nutritional status); the population sub-group (elderly, older adults, ageing, aging); and the exposure (determinants, predictors, risk factors). Figure 1 shows the exact search terms used.

### Inclusion criteria

Studies with populations with mean age > 65 years, majority community-dwelling (> 80%), and conducted in Western populations (specifically European, North American, Canadian, Australian and New-Zealanders) were selected for consideration. Studies containing populations from multiple countries were only included if the majority of the population came from the specified Western countries. As the standardised criteria for diagnosing malnutrition were only published in 2019 [26, 27], papers using any definition of malnutrition arising from use of screening tools, specific BMI cut-offs or weight loss percentages were considered for inclusion. Only papers which were published since 2000, peer-reviewed, available in full-text, written in English, conducted on humans and in which the study authors completed multivariate statistical analysis were considered for inclusion. As the main aim of this review was to assess the sociodemographic factors associated with malnutrition according to a population’s rate of ageing, studies examining a combination of biochemical or nutritional factors in addition to sociodemographic factors were excluded [29–31].

### Study selection

Abstracts were screened for inclusion by two authors independently (LAB and ML). If a study appeared to meet the inclusion criteria, full text articles were read and analysed for inclusion by two authors working independently (LAB and MGB). Final inclusion was decided by consensus discussion with a senior researcher working on the topic of community malnutrition (PDC).

### Data synthesis

**Fig. 2:**
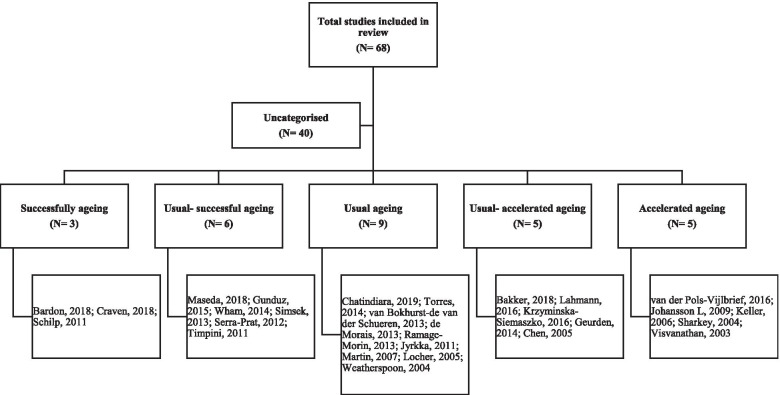
Categorisation of studies included in review by rate of ageing

Selected full-text articles were read in full and the investigated factors categorised into domains. Factors suggested as being associated with malnutrition or as determinants of malnutrition were categorised under nine known domains: demographic, food intake, oral, lifestyle, social, economic, physical functioning, psychological and disease-related [32]. For the purposes of this review, poverty was included in the social domain and both edentulousness and chewing difficulties included in the food intake domain. Factors reported within each domain are summarised in Table 1. Where possible, study populations were categorised into successful, usual or accelerated rate of ageing groups according to the criteria suggested by Keller et al. (2007), as summarised below [15] (Fig. 2).

- Successful ageing: predominantly functionally independent (> 60%), not frail (< 40%), low prevalence of polypharmacy (< 40%), and low prevalence of multi-morbidity (< 40%).
- Usual ageing: predominantly functionally independent (> 60%), not frail (< 40%), a high proportion regularly attending a GP (> 50%), high prevalence of multi-morbidity (> 50%) and polypharmacy (> 50%).
- Accelerated ageing: predominantly frail (> 60%), functionally dependent (> 60%), users of home-care services (> 40%), and a high proportion was recently hospitalised (> 50%).

**Table 1:** Reported associated factors, and determinants, of malnutrition in community-dwelling older adults by domain

Demographic	Food Intake	Lifestyle	Social	Physical function	Psychological	Disease-related
Age [33]	Reduced appetite [34]	No alcohol use [35]	Poverty [36]	Frailty [37]	Depression [38]	Polypharmacy [39]
Marital status [33]	Edentulousness [38]	Smoking status [40]	Living alone [41]	Dependency [40]	Dementia [38]	Chronic disease [42]
Sex [43]	Ability to self-feed [44]	Low physical activity [40]	Social support [45]	Mobility [46]	Cognitive decline [46]	Self-reported health status [37]
Education [42]				Falls [47]	Anxiety [37]	Hospitalisation [46]
				Handgrip strength [43]		Acute disease [40]
						Pain [48]

For each of the parameters listed above, any measure or tool or definition used by a particular study was deemed acceptable. In order for study populations to be categorised, information had to be available for at least two of the above criteria. Study populations were placed between two categories if there was insufficient information to differentiate which specific category the population should be placed in.

## Results

### Search results

**Fig. 3:**
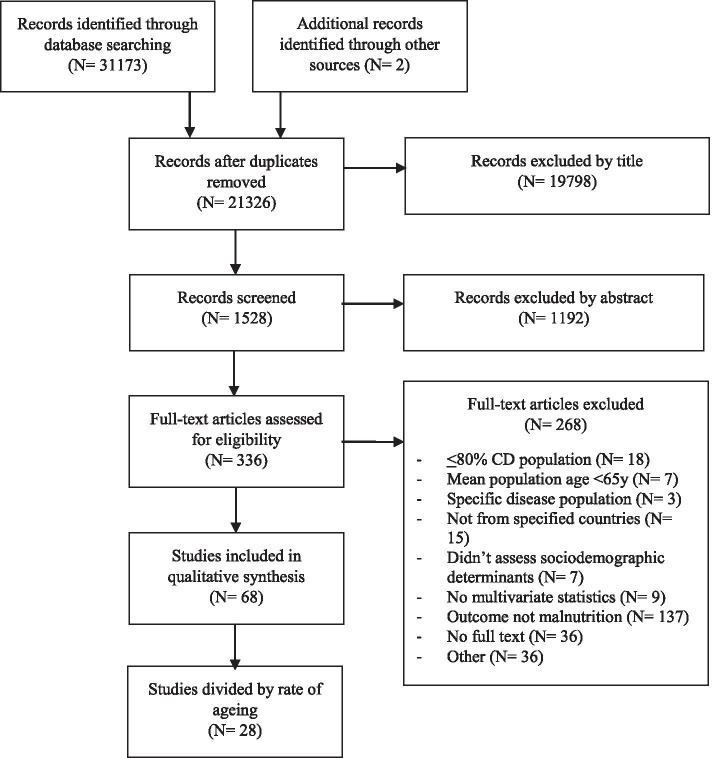
Flow chart of selection criteria for inclusion in review

The initial database search yielded 21,326 papers once duplicates were deleted. All papers were considered for inclusion; reasons for exclusion are outlined in Fig. 3. The most common reasons for exclusion were studies conducted in non-Western populations, younger populations (mean < 65 years), populations with a specific disease or condition (e.g., Parkinson’s disease), studies whose primary focus was not malnutrition and studies completed in hospital, residential care, or rehabilitation settings. A total of 68 papers met the final inclusion criteria (Fig. 3).

The articles included were heterogeneous in study design (Table 2). Studies were predominantly of cross-sectional (*N* = 54) or longitudinal design (*N* = 11). There were two systematic reviews of observational studies and one meta-analysis of longitudinal studies. Sample size of the studies ranged from 49 to 15,669 participants. The majority of included studies were conducted within European countries.

**Table 2:** Factors associated with, and, determinants of malnutrition

First Author, Year, Country, Sample size, Age (mean (SD))	Sex (male %), Setting, Rate of Ageing	Outcome (assessment method)	Domain: Determinants Assessed	FU time	Statistical Analysis	Key Results* [OR (95% CI) ***p***-value]
**Cross-sectional studies**						
Chatindiara [49], 2019, New Zealand, *N* = 257, median 79 (IQR 7)	46.7, CD, U	MN risk (MNA-SF)	Demographic: age, marital status, ethnicity, sex, education Social: Living situation, income source Food Intake: dysphagia risk (EAT-10), dental status Psychological: cognitive impairment (MoCA) Physical function: ADLs, handgrip strength, gait speed, physical performance (FTSTS) Disease-related: inflammation (CRP), number of comorbidities (> 5), polypharmacy (> 5 drugs), nutrition supplements use	N/A	UV LR MV LR	age (continuous) [1.09 (1.01–1.17) *p* = 0.033]; age < 85y [0.30 (0.1–0.79) *p* = 0.015]; normal swallowing [0.29 (0.09–0.97) *p* = 0.045]; healthy physical performance [0.22 (0.07–0.71) *p* = 0.012]; BMI [0.82 (0.74–0.91) *p* < 0.001]; fat mass [0.86 (0.78–0.94) *p* = 0.002]; % body fat [0.81 (0.72–0.90) *p* < 0.001]; FFMI [0.51 (0.34–0.77) *p* = 0.001]
Craven [23], 2018, Australia, *N* = 77, 73.3 (5.1)	60.0, CD, S	MN risk (SCREEN 2)	Demographic: age, sex, relationship status, education Food Intake: SR healthiness of diet Social: living arrangement, home care services Disease-related: SR health, short form health survey (SF-12)- calculated PCS and MCS	N/A	Multiple regression	PCS (ẞ = 0.290, Seẞ = 0.065, *p* < 0.05); MCS (ẞ = 0.377, Seẞ = 0.073, *p* < 0.05)
Maseda [45], 2018, Spain, *N* = 749, 75.8 (7.2)	39.4%, CD attending SC, U-S	MN risk (MNA-SF)	Demographic: age, sex, education, marital status Social: social support (OARS), living situation, loneliness Physical function: IADL Disease-related: QOL (WHOQOL-BREF)	N/A	Multiple LR (forward stepwise)	Total: female sex [0.6 (0.38–0.95) *p* = 0.028], social resources- total impairment [0.257 (0.08–0.85) *p* = 0.025], low physical health [1.676 (1.09–2.57) *p* = 0.018] Males: single status [0.08 (0.02–0.34) *p* < 0.001], divorced/separated status [0.096 (0.02–0.39) *p* < 0.001], poor health satisfaction [4.31 (1.82–10.25) *p* < 0.001] Females: social resources- mild impairment [0.51 (0.28–0.96) *p* = 0.036]
Ganhão-Arranhado [50], 2018, Portugal, *N* = 337, 78.4 (7.05)	37.7%, CD attending SC, N/A	MN, MN risk (MNA)	Demographic: age group, sex, marital status Social: income, SC attendance, motives for SC attendance, time of SC attendance, social risk, social net, social relationships Food Intake: food security Lifestyle: alcohol consumption, smoking status Disease-related: SR health, SR health conditions (respiratory, liver and rheumatic diseases, angina, MI, high BP, high blood cholesterol, stroke, DM, cancer, depression) Psychological: psychological stress	N/A	UV regression, multinomial regression	MN risk: cerebrovascular accident [4.04 (1.19–13.74) *P* < 0.05]; acute MI [2.12 (0.95–4.72) *p* < 0.05]; better perceived health status [− 0.54 (0.37–0.79) *p* < 0.05]; attending SC <5y [− 0.41 (0.16–1.04) *p* < 0.05]; loneliness [2.01 (1.06–3.81) *p* < 0.05] MN: food insecurity [1.73 (1.20–2.48) *p* < 0.05]; female [7.87 (1.33–46.72) *p* < 0.05]; age 74-85y [− 0.10 (0.02–0.57) *p* < 0.05]; depression [37.41 (2.06–679.55) *p* < 0.05]; DM [− 0.105 (0.01–1.06) *p* < 0.05]
Fjell [51], 2018, Norway, *N* = 166, 78.7 (3.3)	42%, CD, N/A	MN risk (MNA-SF)	Demographic: age, sex, education, marital status Social: social support (OSLO 3-SSS) Lifestyle: exercise, alcohol consumption, smoking status Other: vision, hearing, sleep problems Disease-related: SR pain, hypertension, hypercholesterolemia, eye disease, arthrosis, cancer Psychological: depression	N/A	MV LR	poor SR health [5.77 (2.04–16.29) *p* = 0.001]
Grammatikopoulou [52], 2018, Greece, *N* = 207, 72.4 (8.5)	43.5%, CD, N/A	MN risk (MNA)	Demographic: age, education, marital status, waist circumference, BMI Social: income, receiving financial assistance, Lifestyle: smoking status Food intake: appetite (CNAQ), food security (HFIAS), dietary variety (HDDS), diet quality (MEDAS) Disease-related: catabolic disease (cancer/renal/lung), cardiometabolic disease (CVD, hypertension, angina, arrhythmia, hyperuricemia, microalbuminuria, retinopathy, neuropathy, or history of acute MI, stroke or coronary by-pass surgery)	N/A	UV LR MV LR	smoking [2.35 (1.09–5.08) *p* = 0.030]; not being married [2.10 (1.06–4.15) *p* = 0.033]; at risk for 5% WL [7.86 (4.07–15.18) *p* < 0.001]; food insecure [2.63 (1.21–5.75) *p* = 0.015]
Bakker [53], 2018, The Netherlands, *N* = 1325, median (IQR) 80 (77–84)	41.4%, CD attending GP, U-A	MN (BMI < 20 kg/m^2^ and/or unintentional WL > 10% in 6 m and/or unintentional WL > 5% in 1 m)	Demographic: age, sex, marital status, education, income Social: living situation Food intake: oral status, irregular dentist visits, oral hygiene, chewing problems, eating problems, speech problems, dental pain, dry mouth, insecurity with oral status, satisfaction with oral status Physical function: frailty (GFI), risk profile (frail, complex care needs, robust), ADL (Katz-15) Disease-related: number of chronic conditions, polypharmacy (> 4 drugs), complex care (IM-E-SA), QOL (EQ-5D, EQ-VAS)	N/A	UV LR MV LR	health related QOL [0.97 (0.95–0.995) *p* = 0.015]
Jung [54], 2017, America, *N* = 171, 77.5 (8.2)	29.8%, Rural CD excluding mild- moderate dementia (SPMSQ), N/A	MN risk (MNA-SF)	Demographic: age, sex, race or ethnicity, marital status, education Social: annual income, loneliness (UCLA loneliness scale) Psychological: depression (GDS) Disease-related: health status (SHPS), Physical function: ADL, IADL (Self-Care Capacity Scale)	N/A	SEM	parameter estimate (standard error): depression −0.30 (0.10) *p* = 0.001
van der Pols-Vijlbrief [40], 2016, The Netherlands, *N* = 300, 81.7 (7.6)	31.7%, CD, receiving home-care, A	MN risk (SNAQ^65+^)	Demographic: sex, age, education level, marital status Social: living situation, social network (LSNS-6), social support, monthly income, financial ability to buy food (Determine your health checklist) Food intake: eating alone, SR oral health, chewing surface (full vs partial/none) appetite (SNAQ^app^), taste/smell loss, adequate snacks per day (> 3) Lifestyle: smoking status, alcohol consumption, PA Disease-related: number chronic diseases (> 2), polypharmacy (> 5 drugs), hospitalisation in past 6 m, SR health, pain (NHP), nausea, intestinal problems, fatigue Psychological: cognitive decline (IQCODE), depression (CES-D-10) Physical functioning: ADL (BI), IADL, mobility (bed/chair bound, able to move around the house but unable to leave house independently, able to leave house independently, difficulty climbing stairs, ability to walk 100 m), falls Other: visual function, hearing function	N/A	MV LR	unable to go outside [5.39 (2.46–11.81) *p* < 0.001], intestinal problems [2.88 (1.57–5.28) *p* = 0.001], smoking [2.56 (1.37–4.77) *p* = 0.003], osteoporosis [2.46 (1.27–4.76) *p* = 0.007], fewer than 3 snacks per day [2.61 (1.37–4.97) *p* = 0.003], ADL dependency [1.21 (1.09–1.35) *p* = 0.001], physical inactivity [2.01 (1.13–3.59) *p* = 0.018], nausea [2.50 (1.14–5.48) *p* = 0.022], cancer [2.84 (1.12–7.21) *p* = 0.028]
Lahmann [44], 2016, Germany, *N* = 878, 78.5 (12.2)	37.1%, CD home care recipients, U-A	MN risk (MUST, MNA-SF)	Demographic: age, sex Social: social living status Disease-related: duration receiving home care Physical functioning: functional capacity (BI)	N/A	LR	mental overload [8.1 (2.2–30.2) *p* < 0.01]; loss of appetite [3.6 (1.8–7.3) *p* < 0.01]; needs help with feeding [5.0 (2.3–11.2) *p* < 0.01]; dependent on feeding [1.9 (1.2–2.8) *p* < 0.01]
Maseda [55], 2016, Spain, *N* = 749, 75.8 (7.2)	39.4%, CD attending SC, N/A	MN risk (MNA-SF)	Demographic: sex, age, education level, BMI ≥25 kg/m^2^ Disease-related: co-morbidity (CCI), SR health, polypharmacy (> 5 drugs) Psychological: cognitive impairment (MMSE), depressive symptoms (GDS-SF) Physical functioning: frailty status	N/A	muliple LR (forward stepwise likelihood ratio)	Total: BMI > 25 kg/m^2^ [2.15 (1.28–3.61) *p* = 0.004]; polypharmacy [0.43 (0.28–0.68) *p* < 0.001]; poor SR health [0.32 (0.12–0.86) *p* = 0.023]; depressive symptoms [0.45 (0.23–0.86) *p* = 0.015]; pre-frail/frail [0.51 (0.28–0.93) *p* = 0.027] Females: polypharmacy [0.52 (0.31–0.88) *p* = 0.014]; poor SR health [0.24 (0.09–0.66) *p* = 0.005] Males: BMI > 25 kg/m^2^ [4.35 (1.61–11.75) *p* = 0.004]; polypharmacy [0.26 (0.11–0.62) *p* = 0.002]; depressive symptoms [0.10 (0.04–0.31) *p* < 0.001]
Krzyminska-Siemaszko [38], 2016, Poland, *N* = 3751, 77.4 (8.0)	52.8%, CD excluding cognitively impaired (MMSE), U-A	MN risk (MNA-SF)	Psychological: depression (GDS), cognitive impairment (MMSE) Disease-related: polypharmacy (> 5 drugs), number chronic diseases (> 4), anaemia, peptic ulcer, stroke, Parkinson’s, cancer, pain Food intake: edentulism	N/A	Multiple LR	Total: female sex [1.72 (1.45–2.04) *p* < 0.001], age [2.16 (1.80–2.58) *p* < 0.001], depression [11.52 (9.24–14.38) *p* < 0.001], dementia [1.52 (1.20–1.93) *p* < 0.001], multi-morbidity [1.27 (1.04–1.57) *p* = 0.02], anaemia [1.80 (1.41–2.29) *p* < 0.001], total edentulism [1.26 (1.06–1.49) *p* = 0.009] Males: age [1.78 (1.40–2.27) *p* < 0.001], depression [12.80 (9.40–17.43) *p* < 0.001], dementia [1.58 (1.15–2.18) *p* = 0.005], anaemia [1.81(1.34–2.44) *p* < 0.001], total edentulism [1.31(1.04–1.66) *p* = 0.02] Females: age [2.77 (2.11–3.61) *p* < 0.001], depression [10.80 (7.85–14.87) *p* < 0.001], multi-morbidity [1.35 (1.01–1.79) *p* = 0.04], anaemia [1.99 (1.30–3.07) *p* = 0.002]
Krzymińska-Siemaszko [56], 2015, Poland, *N* = 4482, 78.6 (8.5)	52.2%, CD, cognitively well (MMSE), N/A	MN risk (MNA-SF)	Demographic: age, sex, marital status, education Social: living situation	N/A	UV LR MV LR	female [1.51 (1.19–1.92) *p* < 0.01]; every 10 y of life [2.18 (1.9–2.51) *p* < 0.01]; not married [1.50 (1.16–1.95) *p* < 0.01]
Gunduz [42], 2015, Turkey, *N* = 1030, 71.7 (7)	45.05% CD, outpatients, cognitively well (MMSE > 17), U-S	MN (MNA)	Demographic: age, sex, marital status, education, no children Physical functioning: ADL, IADL Psychological: depression (GDS) Disease-related: comorbidities, polypharmacy (≥5 drugs)	N/A	MV LR	age [(1.007–1.056) *p* = 0.012]; low BMI [(0.702–0.796) *p* < 0.001]; low education level [(0.359–0.897) *p* = 0.015]; depression score [(1.104–3.051); *p* = 0.02]; > 4 comorbidities [3.5 (2.30–5.45) *p* < 0.001]
Bailly [57], 2015, France, *N* = 464, 77.41 (7.48)	31.3%, CD, N/A	MN risk (MNA)	Demographic: age Social: living alone, financial satisfaction Food intake: pleasure of eating (HTAQ) Psychological: depressive symptoms (GDS) Disease-related: SR health Physical functioning: IADL	N/A	SEM	Males: depression β = − 0.38; greater pleasure eating β = 0.20; higher SR health β = 0.32; greater IADL score β = 0.16 Females: age β = − 0.13; depression β = − 0.33; greater pleasure eating 0.19; higher SR health β = 0.25; greater IADL score β = 0.32
Wham [58], 2015, New Zealand, Maori: *N* = 421, 82.8 (2.6) Non-maori: *N* = 516, 84.6 (0.5)	33%, CD, N/A	MN risk (SCREEN 2)	Demographic: age, sex Lifestyle: PA (PASE), smoking status, alcohol consumption Social: residential care, living situation, life satisfaction, difficulty getting to shops, drives a car, occupation, deprivation index, income Physical functioning: HGS, physical function (NEADL) Disease-related: health related QOL (SF-12), stroke, MI Psychological: cognitive function (3MS), depression (GDS-15)	N/A	MV LR	Maori: age [0.89 (0.79–0.99) *p* = 0.04]; primary education [3.41 (1.35–8.62) *p* = 0.03]; living alone (vs with others) [2.85 (1.34–6.05) *p* < 0.001]; living alone (vs with spouse) [4.10 (1.90–8.84) *p* < 0.001]; depression [1.30 (1.06–1.60) *p* = 0.01] Non-Maori: male [0.49 (0.30–0.81) *p* = 0.005]; living alone (vs with spouse) [2.41 (1.42–4.08) *p* = 0.002]; SF-12 PCS [0.98 (0.96–0.99) *p* = 0.02]; depression [1.24 (1.08–1.43) *p* = 0.002]
Wham, 2015 [59], New Zealand, *N* = 67, 77.0 (1.5)	44%, CD Maori, N/A	MN risk (SCREEN 2)	Demographic: sex, age, education, marital status Social: living situation, SR standard of living, importance of traditional food, importance of spirituality, use of traditional Māori as first language, living in large extended family area Disease-related: use of Māori medicine and healing Psychological: depression (GDS-15) Physical functioning: physical disability (NEADL)	N/A	MV linear regression	language and culture being a little to moderately important [ẞ = 6.70, *p* < 0.05]; availability of traditional food [ẞ = − 5.23, *p* < 0.01]; waist-to-hip ratio [ẞ = 20.17, *p* = 0.01]; depressive symptoms [ẞ = − 0.60, *p* = 0.02]
Toussaint [60], 2015, The Netherlands, *N* = 345, 67.1 (6.0) CD; *N* = 138, 80.9 (7.6) outpatients	46.4%, CD; 34.1%, outpatients, N/A	MN risk (MNA-SF)	Demographic: age, sex Food intake: olfactory function Lifestyle: smoking status Psychological: cognitive function (CD: MMSE; outpatients: DemTect), depressive symptoms (GDS) Disease-related: comorbidities (CCI), polypharmacy (> 5 drugs)	N/A	Linear regression	CD: female [0.259 (0.031–0.488) *p* = 0.026] Outpatients: MMSE [ẞ (95% CI) *p*-value] [0.208 (0.059–0.357) *p* = 0.007]; GDS [− 0.378 (− 0.491- − 0.265) *p* < 0.001]
Rullier [61], 2014, France, *N* = 56, 70.9 (11.0)	27%, CD caregivers, N/A	MN risk (MNA)	Demographic: age, sex, education, caregiver relationship with patient Social: living arrangements Psychological: Trait anxiety (STAI Y-B), depression (CES-d), caregiver burden (Zarit Burden Interview) Physical functioning: functional status (AGGIR)	N/A	UV linear regression, multiple linear regression	functional dependency [ẞ = − 0.336, (1.57–6.48) *p* = 0.002]; depressive symptoms [ẞ = − 0.365, (− 0.199- − 0.054) *p* = 0.001]; more apathetic patient with dementia [ẞ = − 0.342 (− 0.606- − 0.158) *p* = 0.001]
Torres [39], 2014, France, Rural: *N* = 692, 75.5 (6.2) Urban: *N* = 8691, 74.1 (5.5)	62% (rural), 39.7% (urban), CD, U	MN risk (proxy MNA)	Demographic: age, sex, education, marital status Social: income Physical function: ADL (Katz ADL scale) Disease-related: polypharmacy (> 3 drugs)	N/A	MV LR	Rural: BMI < 21 kg/m^2^ [23.09 (5.1–104.46) *p* < 0.01], BMI 25–30 kg/m^2^ [0.41 (0.18–0.94) *p* < 0.01], BMI > 30 kg/m^2^ [0.16 (0.05–0.50) *p* < 0.01], dementia [3.04 (1.08–8.57) *p* = 0.04], polypharmacy [10.4 (2.59–4.20) *p* < 0.01] Urban: females [1.46 (1.22–1.75) *p* < 0.001], widowed status [1.36 (1.12–1.66) *p* < 0.01], BMI < 21 kg/m^2^ [9.11 (7.39–11.23) *p* < 0.001], BMI 25–30 kg/m^2^ [0.74 (0.61–0.89) *p* < 0.001], depression [20.67 (17.46–24.49) *p* < 0.001], dementia [3.42 (2.22–2.58) *p* < 0.001], loss of ADL [6.94 (3.91–12.31) *p* < 0.001], polypharmacy [3.52 (2.95–4.20) *p* < 0.001]
Wham [41], 2014, New Zealand, *N* = 3893, > 65y, Maori: >75y	46%, CD, U-S	MN risk (ANSI)	Demographic: age, sex, marital status, ethnicity, education Social: WHOQOL- social, living situation Physical functioning: ADLs (NEADL) Psychological: depression (GDS) Disease-related: chronic diseases, polypharmacy (> 3 drugs)	N/A	UV LR MV LR	female [1.41 (1.11–1.80) *p* = 0.006]; being Māori/other ethnicities vs European *p* = 0.002; not married *p* = 0.003; higher social health related QOL [0.94 (0.89–1.00) *p* = 0.036]; living with others related to low risk *p* < 0.0001; higher functional status [0.94 (0.90–0.99) *p* = 0.0182]; more depressive symptoms [1.10 (1.02–1.19)]; polypharmacy [1.34 (1.27–1.41) *p* < 0.0001]
Akin [62], 2014, Turkey, *N* = 845, 71.6 (5.6)	53.2%, urban CD, N/A	MN risk (MNA)	Demographic: sex, age, weight, BMI, WC, MUAC, education, marital status Social: living situation, income Physical functioning: 4 min walking speed, fear of falling, IADL, ADL, urinary incontinence Disease-related: SR chronic diseases (diabetes, hypertension, CHD, cerebrovascular disease, renal failure) Psychological: cognitive impairment (MMSE), depression (GDS)	N/A	UV LR MV LR	depressive mood [4.18 (2.85–6.11) *p* < 0.001]; diabetes [1.60 (1.09–2.35) *p* = 0.017]; moderate income [1.65 (1.08–2.49) *p* = 0.019]; low income [2.36 (1.48–3.77) *p* < 0.001]; living alone [2.49 (1.56–3.97) *p* < 0.001]; WC [0.98 (0.96–0.99) *p* = 0.015]; MUAC [0.93 (0.83–0.99) *p* = 0.014]; 4 min walking speed [1.16 (1.07–1.25) *p* < 0.001]
Geurden [63], 2014, Belgium, *N* = 100, 75.2 (17)	22%, urban CD receiving homecare nursing, U-A	MN risk [64]	Demographic: age, sex Food intake: eating problem, swallowing problem, loss of appetite, concern about eating problem/loss of appetite, GP informed about eating problem/loss of appetite, nutrition intervention prescribed, one warm meal every day Physical functioning: independent shopping, independent cooking, use of informal care, use of professional homecare Disease-related: hospitalisation in last 3 m, days since last GP visit	N/A	MV LR	loss of appetite *p* < 0.001
Westergren [65], 2014, Sweden, *N* = 465, 78.5 (3.7)	46.5%, CD without cognitive deficits, N/A	MN risk (SCREEN 2)	Social: need for help with groceries, need for help with cooking Physical functioning: falls (Downton falls risk index) Disease-related: SR health Psychological: SR life satisfaction, anxiety/worries, low-spiritedness, fatigue/tiredness, sleeping well	N/A	stepwise ordinal regression Linear (backward) regression	living alone (females) [4.63 (2.85–7.52) *p* < 0.001]; living alone (males) [6.23 (3.35–11.59) *p* < 0.001]; age [0.86 (0.81–0.91) *p* < 0.001]; quite good SR health [2.03 (1.27–3.27) *p* = 0.003]; quite/very poor SR health [5.01 (2.23–11.23) *p* < 0.001]; often/always tired [2.38 (1.26–4.50) *p* = 0.008]; falls risk [1.21 (1.05–1.40) *p* = 0.010]
van Bokhorst-de van der Schueren [35], 2013, Netherlands, *N* = 448, 80 (7)	38%, outpatients living independently- in own home or assisted care facility, U	MN (MNA)	Demographic: education, marital status, children Lifestyle: smoking status, alcohol consumption Physical functioning: ADLs, IADLs, falls, walking aid Psychological: depression (GDS), cognitive impairment (MMSE) Disease-related: polypharmacy (> 6 drugs), multi-comorbidities (> 4 diseases)	N/A	UV LR MV backward stepwise LR	alcohol use [0.4 (0.2–0.9) *p* < 0.05]; being IADL dependent [2.8 (1.3–6.4) *p* < 0.05]; depression [2.6 (1.3–5.3) *p* < 0.05]
de Morais [66], 2013, 8 European countries (Denmark, Germany, Italy, Poland, Portugal, Spain, Sweden and the UK), *N* = 644, 74.8 (5.8)	49.8%, CD, U	MN risk (Determine your health checklist)	Demographic: BMI Social: living situation Food Intake: number of fruit and vegetables per day, chooses easy to chew food, changes in appetite Disease-related: SR health, changes in health/health problems (SF-36)	N/A	backward stepwise LR	low BMI [ẞ (95% CI) *p*-value] [0.005 (0.001–0.01) *p* = 0.007]; number fruit and vegetables/day [− 0.21 (− 0.40- -0.03) *p* = 0.023]; general health [− 0.02 (− 0.03- -0.01) *p* = 0.006]; chooses easy to chew food [0.32 (0.15–0.49) *p* < 0.001]; living with another adult [2.82 (1.27–6.25) *p* = 0.011]; living alone [3.22 (2.00–5.16) *p* < 0.001]; changes in appetite [0.41 (0.20–0.85) *p* = 0.016]; changes in health/health problems [7.74 (4.02–14.90) *p* < 0.001]
Syrjälä [67], 2013, Finland, *N* = 157, > 75y	29.9%, CD, N/A	MN risk (MNA-SF)	Demographic: sex, education Food Intake: unstimulated salivary flow, stimulated salivary flow, number of teeth, number of the occluding molars/pre-molars, dentures, SR chewing problems Social: use of a meal service Disease-related: number of medications, DM Psychological: cognitive function (MMSE) Physical functioning: IADLs	N/A	MV LR	Stimulated/unstimulated salivary flow not associated with MN risk
Simsek [68], 2013, Turkey, *N* = 650, 74.1 (6.3)	37.1%, CD living in a low socioeconomic area, U-S	MN risk (MNA)	Demographic: age, sex, marital status, education Social: self-perceived economic status, social class, social insurance, ownership of house, personal income, living situation Food intake: food insecurity Physical functioning: orthopaedic disability Disease-related: number chronic diseases, polypharmacy (> 5 drugs), SR health	N/A	MV LR	age [1.06 (1.02–1.10) *p* = 0.001]; number chronic diseases [1.41 (1.18–1.70) *p* < 0.001]; not being married [2.13 (1.31–3.46) *p* = 0.002]; SR poor economic status [2.49 (1.41–4.41) *p* = 0.002]; orthopaedic disability [1.95 (1.01–3.75) *p* = 0.047]; food insecurity [2.49 (1.48–4.16) *p* = 0.001]; poor SR health [4.33 (2.58–7.27) *p* < 0.001]
Smoliner [69], 2013, Germany, *N* = 191, 79.6 (6.3)	28.3%, CD day hospital attendees without Parkinson’s disease or MMSE score < 20, N/A	MN risk (MNA)	Demographic: age Food intake: olfactory function (Sniffin sticks test) Psychological: cognitive function (MMSE) Disease-related: number of drugs Physical functioning: self-care capacity (BI)	N/A	Linear regression	BI [0.329 (0.03–0.08) *p* < 0.001]
Ramage-Morin [70], 2013, Canada, *N* = 15,669, 77 (No SD)	40.4%, CD, U	MN risk (SCREEN 2-AB)	Demographic: age, education Food Intake: oral health Social: income quintile, living situation, social support (Tangible Support MOS Subscale), social participation, driving status Disease-related: number of medications Psychological: depressive symptoms (subset of questions from CIDI) Physical functioning: level of disability (HUI)	N/A	MV LR	Males: lowest income quintile [1.46 (1.16–1.85) *p* < 0.05]; living alone [2.86 (2.39–3.42) *p* < 0.05]; low social support [1.31 (1.06–1.62) *p* < 0.05]; infrequent social participation [1.46 (1.20–1.76) *p* < 0.05]; depression [2.77 (1.51–5.06) *p* < 0.05]; moderate/severe disability [1.59 (1.32–1.90) *p* < 0.05]; taking 2–4 drugs/day [1.31 (1.10–1.56) *p* < 0.05]; taking > 5 drugs/day [1.69 (1.17–2.44) *p* < 0.05] Females: age [0.98 (0.97–0.99) *p* < 0.05]; living alone [1.85 (1.61–2.12) *p* < 0.05]; low social support [1.49 (1.26–1.75) *p* < 0.05]; infrequent social participation [1.43 (1.22–1.69) *p* < 0.05]; depression [2.21 (1.54–3.17) *p* < 0.05]; moderate/severe disability [1.82 (1.58–2.11) *p* < 0.05]; 2–4 drugs/day [1.42 (1.23–1.63) *p* < 0.05]; > 5 drugs/day [2.23 (1.71–2.91) *p* < 0.05]; fair/poor oral health [1.54 (1.27–1.88) *p* < 0.05]
Söderhamn [71], 2012, Norway, *N* = 2106, 74.5 (6.9)	49.5, CD, N/A	MN risk (NUFFE-NO, MNA-SF)	Demographic: age, sex, marital status Lifestyle: being active Food Intake: eating sufficiently, preparing food, having access to meals Social: occupation, social support (receiving help to manage daily life), frequency of contact with family/neighbours/friends, loneliness, receiving home nursing, receiving home help Disease-related: SR health, presence of chronic disease/handicap Psychological: feeling depressed	N/A	UV LR MV LR (forward stepwise conditional)	NUFFE-NO: single [2.99 (2.17–4.13) *p* < 0.001]; professional/white collar worker [0.50 (0.36–0.69) *p* < 0.001]; depressed [1.71 (1.07–2.76) *p* = 0.026]; chronic disease/handicap [2.15 (1.57–2.96) *p* < 0.001]; being active [0.26 (0.17–0.39) *p* < 0.001]; eating sufficiently [0.07 (0.02–0.21) *p* < 0.001]; receiving home nursing [2.99 (1.37–6.56) *p* < 0.006]; receiving family help [1.92 (1.40–2.64) *p* < 0.001]; contact with neighbours [0.73 (0.61–0.89) *p* = 0.001] MNA-SF: female [1.70 (1.18–2.43) *p* = 0.004]; receiving help [1.67 (1.02–2.75) *p* = 0.042]; perceived helplessness [2.39 (1.41–4.02) *p* = 0.001]; chronic disease/handicap [1.56 (1.08–2.25) *p* = 0.019]; eating sufficiently [0.18 (0.08–0.39) *p* < 0.001]; receiving home help [1.88 (1.25–2.81) *p* = 0.006]; receiving family help [1.88 (1.25–2.81) *p* = 0.002]; having contacts with family [0.59 (0.40–0.86) *p* = 0.006]; having contacts with neighbours [0.76 (0.62–0.93) *p* = 0.008]
Nykänen [72], 2012, Finland, *N* = 696, 81 (4.6)	30.6%, CD, N/A	MN risk (MNA-SF)	Demographic: age, sex, education Food Intake: dry mouth/chewing problems Social: living situation Disease-related: SR health, number of drugs used regularly Psychological: depressive symptoms (GDS), cognitive impairment (MMSE) Physical functioning: ADLs, IADLs, ability to walk 400 m independently	N/A	UV regression MV regression (stepwise, forward selection)	dry mouth/chewing problems [2.01 (1.14–3.54) *p* < 0.05]; IADL [0.85 (0.75–0.96) *p* < 0.05]; MMSE [0.90 (0.85–0.96) *p* < 0.05]
Tomstad [73], 2012, Norway, *N* = 158, 73.2 (6.9)	41.8%, CD, N/A	MN risk (NUFFE)	Demographic: age, marital status Physical functioning: self-care (SASE) Social: attitude to life (SOC), living situation, social support, receiving home help, perceived helplessness Lifestyle: being active Psychological: perceiving life as meaningful	N/A	MV LR (forward stepwise conditional)	living alone [7.46 (2.58–21.53) *p* < 0.001]; receiving help regularly [9.32 (2.39–36.42) *p* = 0.001]; being active [0.17 (0.04–0.65) *p* = 0.010]; perceived helplessness [6.87 (1.44–32.78) *p* = 0.016]
McElnay [74], 2012, New Zealand, *N* = 473, 74.0 (no SD)	43.8%, CD, N/A	MN risk (SCREEN 2)	Demographic: ethnicity (Maori vs not), sex, age Social: living situation	N/A	UV LR MV LR (model 1, forced entry; model 2, forward stepwise)	Model 1: Maori [5.21 (1.52–17.90) *p* = 0.009]; living alone [3.53 (2.06–6.06) *p* < 0.001] Model 2: Maori [6.44 (1.87–22.11) *p* = 0.003]
Zeanandin [75], 2012, France, *N* = 190, 81.2 (4.4)	37.4%, CD, N/A	MN risk (MNA-SF)	Demographic: BMI Food intake: restrictive diet type, diet duration, diet compliance Disease-related: comorbidities, polypharmacy	N/A	UV LR MV LR	absence of diet [0.3 (0.1–0.6) *p* < 0.001]; increased BMI [1.3 (1.2–1.5) *p* < 0.001]; on a restrictive diet [3.6 (1.8–7.2) *p* < 0.001]
Samuel [76], 2012, America, *N* = 679, 74.06 (2.8)	0%, CD, N/A	MN risk (MNA-SF)	Demographic: age, race, marital status, education Social: financial strain, annual income, participation in food stamps program, difficulty driving Disease-related: congestive heart failure, cancer	N/A	MV LR	Enough to make ends meet model: not enough to make ends meet [4.08 (1.95–8.52) *p* < 0.05]; income < $6000/m [2.54 (1.07–5.99) *p* < 0.05]; age [1.12 (1.03–1.22) *p* < 0.05] Lack of income for food model: lack of money fairly/very often [2.98 (1.15–7.73) *p* < 0.05]; income < $6000/m [2.77 (1.10–6.98) *p* < 0.05]; age [1.11 (1.02–1.21) *p* < 0.05]
Timpini [77], 2011, Italy, *N* = 698, 75.6 (6.4)	41.5%, CD, U-S	MN risk (MNA-SF)	Demographic: education Lifestyle: PA	N/A	UV LR MV LR models	low education [2.9 (1.2–6.8) *p* < 0.05]; lack of PA- model 1 [4.5 (2.2–9.8) *p* < 0.05], model 2 [4.8 (1.9–11.8) *p* < 0.05]
Kvamme [78], 2011, Norway, *N* = 3111, 71.6 (5.45)	50%, CD, N/A	MN, MN risk [64]	Psychological: anxiety and depression (SCL-10)	N/A	LR	anxiety/depression symptoms with MN risk: males [3.9 (1.7–8.6) *p* < 0.05], females [2.5 (1.3–4.9) *p* < 0.05]
Fagerstrom [79], 2011, Sweden, *N* = 1230, 76.1 (9.9)	42.4%, CD, N/A	MN (BMI < 23 kg/m^2^)	Demographic: age, sex, living arrangement Psychological: cognitive impairment (MMSE) Physical functioning: ADLs	N/A	UV LR MV LR (backward likelihood ratio stepwise)	age [1.02 (1.00–1.04) *p* = 0.032]; being female [2.20 (1.55–3.11) *p* < 0.001]; moderate/severe cognitive impairment [3.32 (1.77–6.24) *p* < 0.001]
Wham [80], 2011, New Zealand, *N* = 51, 82.4 (1.7)	29.0%, CD, N/A	MN risk (SCREEN 2)	Demographic: age, sex, ethnicity Social: living situation, access to a car, socioeconomic deprivation, strength of social support/network (PANT), loneliness (EASY-Care) Psychological: depression (EASY-Care), cognitive impairment (EASY-Care) Physical functioning: disability score (EASY-Care) Disease-related: SR health (EASY-Care),	N/A	multiple linear regression	good SR health [coefficient (SE) *p*-value] [− 4.31 (1.98) *p* = 0.035]; poor SR health [− 10.23 (2.31) *p* < 0.001]; British/Canadian country of origin [− 5.55 (2.14) *p* = 0.013]; change in living situation, previously with spouse [− 5.31 (2.2) *p* = 0.02]; good SR health*some evidence depression [12.40 (5.24) *p* = 0.023]; poor SR health*some evidence depression [14.96 (5.84) *p* = 0.014]
Romero-Ortuno [81], 2011, Ireland, *N* = 556, 72.5 (7.1)	30.2%, CD independently mobile (with/without walking aid) outpatients, N/A	MN risk (MNA)	Demographic: age, sex Social: social support (LSNS-18), deprivation scale (NIDS), personality traits (EPI), loneliness (De Jong gierveld) Physical functioning: mobility (TUG) Disease-related: comorbidities (CCI) Psychological: cognitive function (MMSE), depressive symptoms (CES-d)	N/A	MV LR	TUG [1.11 (1.05–1.18) *p* < 0.001]; LSNS-18 [0.96 (0.93–0.99) *p* < 0.005]; NIDS [1.20 (1.03–1.39) *p* < 0.018]; age [1.07 (1.01–1.13) *p* < 0.032]
Soderhamn [82], 2010, Sweden, *N* = 1461, > 75 y	45.2, 98% CD, 2% institutionalised, N/A	MN risk (NUFFE)	Demographic: sex, marital status, education Social: living setting Physical function: help to manage daily life Disease-related: perceived health	N/A	MV stepwise LR	living alone [4.85 (3.59–6.56) *p* < 0.05]; receiving help to manage daily life [2.55 (1.77–3.66) *p* < 0.05]; perceived health [0.96 (0.96–0.97) *p* < 0.05]
Sorbye [48], 2008, 11 European sites (Czech Republic, Denmark, Finland, France, Italy, Iceland, Norway, Sweden, Netherlands, Germany, UK), *N* = 4010, 82.5 (7.3)	26%, CD receiving home care or nursing care services, N/A	unintentional WL (> 5% in past 30 days or > 10% in past 180 days)	Demographic: age, sex, severe MN Food intake: < 1 meal/day, insufficient food and fluid intake, insufficient fluid intake, oral problems with swallowing food, pain in the mouth while eating, dry mouth, tube feeding, reduced appetite, vomiting Disease-related: constipation, diarrhoea, daily pain, pain disrupts normal activity, pressure ulcers, SR health, terminal prognosis < 6 m Physical functioning: fall last 90 days, IADL dependency > 3 (index 0–7), ADL dependency > 3 (index 0–8) Other: vision decline past 90 days Social: reduced social activity, feels lonely, not out of house in last week Psychological: risk of depression ≥1 (index 0–9), cognition [CPS > 3 (hierarchy scale 0–6)]	N/A	MV LR (Wald forward stepwise)	< 1 meal/day [4.2 (2.8–6.4) *p* < 0.05]; reduced appetite [2.5 (1.9–3.4) *p* < 0.05]; severe MN [7.1 (4.2–11.9) *p* < 0.05]; reduced social activity [2.0 (1.6–2.5) *p* < 0.05]; hospitalisation past 90 days [2.1 (1.6–2.7) *p* < 0.05]; eating less [2.8 (1.8–4.4) *p* < 0.05]; constipation [1.9 (1.3–2.7) *p* < 0.05]; falls [1.5 (1.2–1.9) *p* < 0.05]; oral problem swallowing food [2.8 (1.8–4.4) *p* < 0.05]; flare-up of chronic disease [1.5 (1.1–2.1) *p* < 0.05]; pressure ulcers [1.5 (1.2–1.9) *p* < 0.05]; daily pain [1.3 (1.0–1.6) *p* < 0.05]
Gil-Montoya [83], 2008, Spain, *N* = 2860, 73.6 (6.8)	42, 88.5% CD, 11.5% institutionalised, N/A	MN risk (MNA)	Demographic: age, sex, institutionalization Food intake: dental status, oral health QOL (GOHAI score)	N/A	multiple linear regression	age [(1.01–1.04) *p* < 0.001]; male [(1.19–1.66) *p* < 0.001]; institutionalisation [(1.16–1.92) *p* < 0.05]; GOHAI [(0.93–0.95) *p* < 0.001]
Roberts [37], 2007, Canada, *N* = 839, 79.6 (no SD)	31.3%, CD with no more than MCI, N/A	MN risk (ENS)	Demographic: sex, age, education, marital status Social: living situation Physical functioning: physical limitations (walking), ADLs/IADLs ^a^ Psychological: cognitive impairment (MMSE) Disease-related: chronic diseases (CDS), SR health status	longitudinal subset (*N* = 335 at risk at baseline): 1 y FU	Cross-sectional: simple LR, MV Longitudinal: simple LR, MV	Cross-sectional: age [1.05 (1.00–1.09) *p* < 0.05]; ADL [1.59 (1.02–2.49) *p* < 0.05]; IADL ‘need’ [1.45 (1.02–2.07) *p* < 0.05]; psychological distress (feelings of depression, anxiety, irritability, impaired cognition) [2.24 (1.22–4.09) *p* < 0.05]; current SR health [3.34 (2.01–5.54) *p* < 0.05] Longitudinal: SR health among those at low MN risk [OR = 3.30, *p* < 0.05]
Martin [84], 2007, America, *N* = 130, 78 (2.3)	45.4%, CD attending VA outpatient clinics with BMI < 24 kg/m^2^, without dementia (MMSE)/ cancer/heart failure, U	MN (BMI < 19 kg/m^2^)	Demographic: age, sex, ethnicity, marital status, education, religion Social: annual income, social support, type of residence Lifestyle: PA, smoking status, alcohol consumption Disease-related: medication use, comorbidities, hospitalisation, doctor visits Psychological: depression (GDS)	N/A	MV LR	having an illness/condition which changed the type/amount of food eaten [4.7 (1.6–13.1) *p* < 0.05], unintentional WL of > 10 lb. in past 6 m [4.0 (1.5–10.7) *p* < 0.05], requiring assistance with travel [4.0 (1.4–11.3) *p* < 0.05]
Chen [36], 2005, America, *N* = 240, 81.7 (8.7)	21.7, CD, U-A	MN risk (MNA)	Demographic: age, sex, marital status, ethnicity, education, religion Social: living situation, income levels, social support (SSQSF), loneliness (UCLA Loneliness Scale) Disease-related: comorbidities (Co-morbidity checklist), medication use Food Intake: oral health (BOHSE, GOHAI) Psychological: depression (GDS) Physical functioning: physical and social competence (ESDS)	N/A	MV hierarchical LR	annual income > $10,000 [0.40 (0.19–0.84) *p* = 0.014], depression [1.12 (1.03–1.21) *p* = 0.008], functional status [1.09 (1.03–1.15) *p* = 0.005], self-perceived oral health [0.87 (0.78–0.97) *p* = 0.009]
Locher [85], 2005, America, *N* = 1000, 75.3 (no SD)	50.1%, CD, U	MN risk (Determine your health checklist)	Demographic: age, education, marital status Social: rural location, income, reliable transportation, social support, y at address, religious attendance, fear attack, experience discrimination, veteran Physical functioning: mobility (Independent life-space)	N/A	multiple linear regression	Black women: reliable transportation [ẞ = 0.196, t = 2.896, *p* = 0.004]; independent life-space [ẞ = − 0.344, t = − 4.626, *p* < 0.001]; income [ẞ = − 0.185, t = − 2.227, *p* = 0.027] Black men: independent life-space [ẞ = − 0.245, t = − 3.415, *p* = 0.001]; being married [ẞ = − 0.245, t = − 3.415, *p* = 0.001]; religious attendance [ẞ = − 0.185, t = − 2.781, *p* = 0.006]; fear attack [ẞ = 0.143, t = 2.300, *p* = 0.023]; experienced discrimination [ẞ = 0.157, t = 2.450, *p* = 0.015] White women: independent life-space [ẞ = − 0.297, t = − 4.121, *p* < 0.001]; social support scale [ẞ = 0.156, t = 2.425, *p* = 0.016]; income [ẞ = − 0.216, t = − 2.259, *p* = 0.025] White men: reliable transportation [ẞ = 0.195, t = 2.957, *p* = 0.003]; independent life-space [ẞ = − 0.282, t = − 4.151, *p* < 0.001]
Johnson [86], 2005, Canada, *N* = 54, 81 (no SD)	48%, CD, N/A	MN risk (MNA)	Social: perceived social support (LSNS) Psychological: life satisfaction (13-item Life Satisfaction Index Form Z), depression (GDS)	N/A	Hierarchical regression analysis (forward selection)	depression (B = − 0.534, *p* = 0.001), social support (B = 0.310, *p* = 0.013)
Weatherspoon [87], 2004, America, *N* = 324, > 60 y (no mean)	25%, CD, U	MN risk (Determine your health checklist)	Demographic: age, sex, ethnicity Social: use of home health aide/caregiver Disease-related: SR health, frequency of doctor, clinic and dentist visits, use of visiting nurse, number of nutritionist/dietitian visits, intake of laxatives, sleep medication, tranquilizers, antacids Food intake: intake of vitamins, fibre supplements, fluid intake	N/A	MV LR	rural location [2.70 (1.2–5.9) *p* = 0.01]; poor SR health [4.28 (1.02–17.9) *p* = 0.04]; not visiting GP regularly [0.34 (0.15–0.77) *p* = 0.01]
Sharkey [88], 2004, America, *N* = 908, 78.2 (8.2)	37.8%, CD MOW recipients, A	MN risk (Nutritional Health Screen- modified version of Determine your health checklist)	Demographic: age, sex, ethnicity (Mexican-American vs not), marital status Social: rural area of residence, poverty guideline Disease-related: multi-comorbidities (> 3 comorbidities) Physical-functioning: ADLs, IADLs	N/A	MV LR	Mexican-American [1.47 (1.05–2.06) *p* = 0.026]; rural [1.49 (1.02–2.18) *p* = 0.04]; not being married [1.77 (1.33–2.36) *p* = 0.001]; worst IADL score [0.44 (0.27–0.70) *p* = 0.001]; worst ADL score [1.74 (1.12–2.71) *p* = 0.014]
Margetts [89], 2003, UK, *N* = 1632, > 65 y (no mean given)	50.7%, CD: 82.5%, institutionalised: 17.5%, N/A	MN risk (MAG tool: high risk = BMI < 18.5 kg/m^2^ or BMI 18.5–20.0 kg/m^2^ with WL of > 3.2 kg or BMI > 20.0 kg/m^2^ with WL > 6.4 kg; medium risk = BMI 18.5–20.0 kg/m^2^ with < 3.2 kg (unless no long-term illness and no WL) or BMI > 20 kg/m^2^ and WL 3.2–6.4 kg; low risk = BMI > 20 kg/m^2^ with no WL (< 5% BW)	Demographic: age, region, setting Disease-related: SR health, long standing illness, hospitalisation in the last y	N/A	MV LR	Males: hospitalisation in past y [1.83 (1.06–3.16) *p* < 0.05]; being institutionalised [2.17 (1.22–3.88) *p* < 0.05]; longstanding illness [2.34 (1.20–4.58) *p* < 0.05]; age > 85 y [2.64 (1.30–5.33) *p* < 0.05]; from northern England/Scotland vs southeast England/London [2.81 (1.54–5.11) *p* < 0.05] Females: poor SR health [2.82 (1.25–6.38) *p* < 0.05]; longstanding illness [2.98 (1.58–5.62) *p* < 0.05]
Sharkey [90], 2002, America, *N* = 729, 79 (no SD)	0%, CD receiving MOW, N/A	MN risk (Determine your health checklist)	Demographic: age, race Social: living situation, income, MOW service use Physical functioning: functional disability (ADL)	N/A	UV ordered LR MV ordered LR	Total sample: being black [coefficient, *p*-value] [0.62, *p* < 0.001]; age 60–74 y [0.80, *p* < 0.001]; poverty [0.43, *p* < 0.001]; living alone [0.51, *p* < 0.001]; increasing m using MOW service [0.096, *p* < 0.05] Black women: aged 60–74 y [0.72, *p* < 0.01] White women: living alone [0.76, *p* < 0.001]; poverty [0.47, *p* < 0.05], aged 60–74 y [0.86, *p* < 0.001]
Pearson [91], 2001, towns within 9 European countries (Belgium, Denmark, France, France, Italy, the Netherlands, Portugal, Spain, Switzerland, Poland), *N* = 627, 80–85 y (no mean/SD given)	45.9%, CD, N/A	MN risk (MNA)	Demographic: sex, living situation Psychological: cognitive impairment (MMSE) Physical functioning: ability to complete all self-care ADLs	N/A	MV LR	Total: diminished cognitive function [2.10 (1.98–2.22) *p* < 0.05]; diminished self-care ability [2.44 (2.32–2.56) *p* < 0.001] Males: diminished self-care ability [2.93 (2.76–3.10) *p* < 0.01]; diminished cognitive function [2.65 (2.46–2.84) *p* < 0.05]; living alone [1.23 (1.06–1.40) *p* < 0.05] Females: diminished self-care ability [2.06 (1.90–2.22) *p* < 0.05]; diminished cognitive function [1.77 (1.61–1.93) *p* < 0.05]
**Longitudinal studies**						
Bardon [46], 2018, Ireland, *N* = 1841, 72 (4.99)	49.8%, CD dementia free (MMSE), S	MN (BMI < 20 kg/m^2^ or WL > 10% over 2 y)	Demographic: age, sex, education, marital status Food intake: appetite Lifestyle: smoking status, alcohol consumption, PA Social: living situation, social support Disease-related: number chronic disease (> 2), polypharmacy (> 5 drugs), pain, SR health, hospitalisation 1 y before baseline, hospitalisation 1 y before FU Physical functioning: falls 1 y before baseline, falls during FU, difficulty climbing stairs without rest, difficulty walking 100 m without rest, HGS Psychological: depression (CES-D), cognitive impairment (MMSE)	2 y	UV LR MV LR	Total: unmarried/separated/divorced status [1.84 (1.21–2.81) *p* < 0.05], hospitalisation 1 y before FU [1.62 (1.14–2.30) *p* < 0.05], difficulty climbing stairs [1.56 (1.12–2.17) *p* < 0.05], difficulty walking 100 m [1.83 (1.13–2.97) *p* < 0.05] Males: falls during FU [1.62 (1.01–2.59) *p* < 0.05], difficulty climbing flight stairs [2.25 (1.44–3.50) *p* < 0.05], hospitalisation 1 y before FU [1.73 (1.08–2.77) *p* < 0.05] Females: social support [2.44 (1.19–4.99) *p* < 0.05], cognitive impairment [2.29 (1.04–5.03) *p* < 0.05]
Hengeveld [92], 2018, America, *N* = 2212, 74.6 (2.9)	49.6%, well functioning CD, N/A	MN (PEM: BMI < 20 kg/m^2^ and/or involuntary WL ≥5% in the past y)	Demographic: age, sex, race, education, BMI Social: living situation, income Lifestyle: PA, smoking status, alcohol consumption Food Intake: diet quality (HEI), protein intake (g/kg BW/d), appetite, biting/chewing difficulty Disease-related: SR health status, chronic diseases (cancer, DM, CVD, chronic pulmonary disease, osteoporosis) Psychological: cognitive function (modified MMSE), depression	yearly for 4 y	MV Cox proportional hazards analysis	Developing PEM during 4 y of FU: low energy intake [0.71 (0.55–0.91) *p* < 0.05] Having persistent PEM (PEM at 2 consecutive FUs): poor HEI score [0.97 (0.95–0.99) *p* < 0.05]; low EI [0.56 (0.36–0.87) *p* < 0.05]; low protein intake [1.15 (1.03–1.29) *p* < 0.05]
Serra-Prat [93], 2012, Spain, *N* = 254, 78.2 (5.6)	53.5%, CD, U-S	MN risk (MNA)	Demographic: age Food Intake: impaired efficacy of swallow (impaired labial seal, oral or pharyngeal residue, piecemeal deglutition) Physical functioning: functional capacity (BI)	1 y	MV LR	No significant results when adjusted for age
Schilp [94], 2011, Netherlands, *N* = 1120, 74.1 (5.7)	48.5%, CD (97.9%) and institutionalised (2.1%), S	MN (BMI < 20 kg/m^2^ or SR invol. WL ≥ 5% in previous 6 m)	Demographic: sex, age, education Food Intake: appetite, chewing difficulties Lifestyle: smoking status, alcohol consumption, PA (LAPAQ) Social: loneliness Physical functioning: limitations climbing stairs, physical performance (chair stands, tandem stand and walk test) Psychological: cognitive impairment (MMSE), depression (CESD), anxiety (HADS) Disease-related: medication use, SR pain, chronic diseases	3, 6, 9 y	cox proportional-HR UV LR MV LR	poor appetite [1.63 (1.02–2.61) *p* < 0.05]; difficulties climbing stairs (in those < 75 y only) [HR (95% CI) 1.91 (1.14–3.22)]; one or two medications (vs none in females only) [HR (95% CI) 0.39 (0.18–0.83) *p* < 0.05]
Jyrkka [78], 2011, Finland, *N* = 294, 81.3 (4.5)	31%, CD (94.6%) and institutionalised (5.4), U	MN risk (MNA-SF)	Demographic: age, sex, education, residential status (home vs institution) Physical function: functional comorbidity index Disease-related: polypharmacy, SR health	3 y	Linear mixed model approach	excessive polypharmacy 0.62 points lower MNA-SF scores (*p* < 0.001); age [ẞ (standard error) *p*-value; − 0.04 (0.02) *p* = 0.016]; institution [− 1.89 (0.25) *p* < 0.001]; moderate [− 0.27 (0.11) *p* = 0.016] and poor [− 1.05 (0.17) *p* < 0.001] SR health status
Johansson Y [43], 2009, Sweden, *N* = 579, 75 y and 80 y cohort	52.5%, CD, N/A	MN risk (MNA)	Demographic: age Physical functioning: HGS, physical mobility (NHP), walking limitations, limitations climbing stairs, physical health (PGC-MAI) ^b^ Psychological: depression (GDS), cognitive impairment (MMSE) Disease-related: SR health, pain (NHP)	75 y olds: yearly for 5 y 80 y olds: yearly for 3 y	forward stepwise multiple LR	higher age *p* = 0.005 at 1 y; HGS [0.938 (0.91–0.97) *p* < 0.001]; physical health [0.65 (0.55–0.76) *p* < 0.001] predicted risk of MN at baseline; more depression symptoms [1.178 (1.07–1.30) *p* = 0.001] 1 y predictor; depressive symptoms*males [OR 1.26] depressive symptoms*females [OR 1.03]; lower SR health [0.432 (0.27–0.70) *p* = 0.001]
Johansson L [95], 2009, Sweden, *N* = 258, 74.2 (2.55)	49.6%, CD, A	MN risk (MNA)	Social: social support Physical functioning: ADLs Psychological: cognitive impairment (MMSE) Disease-related: SR health, hospitalisation	4, 8, 12 y	UV LR MV LR	Total: MOW use [OR 19.6, *p* < 0.001]; Males; use of MOW [OR 21.9, *p* < 0.01]; MMSE score (cut-off 23/24) [12.9 (2.9–56.7) *p* < 0.01]; poorer SR health compared to 4 y ago [OR 5.1, *p* < 0.05] Females: increased MOW use [OR 31.0, *p* < 0.01]; hospital stay during the past 2 m [OR 7.1, *p* < 0.05]
Keller [96], 2006, Canada, *N* = 367, 78.7 (8.0)	24%, vulnerable CD ^c^, A	MN risk (SCREEN)	Social: social support- MOW use	1.5 y	multiple linear regression	MOW use associated with a 1.6-point higher score in SCREEN at FU [(0.02–3.23) *p* = 0.04]; increasing help making meals [(2.91–0.49) *p* = 0.006]
Visvanathan [47], 2003, Australia, *N* = 250, 79.45 (no SD)	30.8%, CD receiving domiciliary care (all had MMSE > 24), A	MN risk (MNA)	Demographic: age Lifestyle: smoking status Social: MOW use, amount of domiciliary care per m Disease-related: comorbidities, health status and QOL (SF-36)	1 y	UV LR binomial analysis	hospitalisation [RR 1.51 (1.07–2.14) *p* = 0.015], > 2 emergency hospitalisation [RR 2.96 (1.17–7.50) *p* = 0.022], > 4 week hospitalisation [RR 3.22 (1.29–8.07) *p* = 0.008], falls [RR 1.65 (1.13–2.41) *p* = 0.013], WL [RR 2.63 (1.67–4.15) *p* < 0.001], > 2 hospitalisations [RR 2.11 (1.04–4.29) *p* = 0.039], emergency hospitalisation [RR 1.99 (1.28–3.11) *p* = 0.002]
Shatenstein [86], 2001, Canada, *N* = 584, > 70 y (no mean given)	40.4%, CD, N/A	MN risk (WL > 5% baseline weight)	Demographic: age, study region, WL Social: ability to shop, bereavement Psychological: cognitive diagnosis at FU, depression, SR interest in life Food Intake: ability to eat independently, loss of appetite Physical functioning: frailty	5 y	MV LR (backward stepwise)	consistent appetite [0.22 (0.12–0.42) *p* < 0.001]; loss of interest in life [0.56 (0.34–0.90) *p* = 0.017]
Ritchie [97], 2000, America, *N* = 563, Males: 77.3 (4.7), Females: 78.1 (5.3)	43%, CD, N/A	MN (WL > 10% BW)	Demographic: age, sex Lifestyle: smoking status, alcohol consumption, PA Food Intake: edentulousness, wears full prostheses, % sites with gingival bleeding, mean attachment loss, mean recession Disease-related: > 2 comorbidities Physical functioning: ADLs Psychological: depression	1 y	UV LR MV LR	female sex [3.77 (1.71–8.33) *p* < 0.05], baseline weight [1.02 (1.01–1.03) *p* < 0.05]; dependent in > 1 ADL [2.27 (1.08–4.78) *p* < 0.05]; edentulousness [2.03 (1.05–3.96) *p* < 0.05]
**Systematic Review**						
O’Keeffe [98], 2018, Canada, Denmark, Finland, Israel, Japan, Netherlands, Spain, Sweden, Taiwan, USA, 23 studies *N* = 108–4512, 74 (12)	17–53.5%, *N* = 15 CD, *N* = 3 institutionalised, *N* = 3 acute hospital, *N* = 2 CD and institutionalised combined, N/A	MN (any definition/screening tool)	Food intake: appetite, complaints about taste, nutrient intake/modified texture diet, hunger, thirst, dental status, chewing, mouth pain, gum issues, swallowing, eating dependency/difficulty feeding Psychological: cognitive function, depression, psychological distress, anxiety Social: social support, living situation, transport, loneliness, wellbeing, MOW, vision and hearing Disease-related: medication use, polypharmacy, hospitalisation, comorbidities, constipation, SR health Physical functioning: ADLs Lifestyle: smoking status, alcohol consumption, PA	24 weeks- 12 y	Mixed	Moderate evidence for association: hospitalisation, eating dependency, poor SR health, poor physical function, poor appetite Moderate evidence for no association: chewing difficulties, mouth pain, gum issues, comorbidity, hearing and vision impairments, smoking, alcohol consumption, low PA, complaints about taste of food, specific nutrient intakes Low evidence determinants: modified texture diets, loss of interest in life, MOW access Low evidence not determinants: psychological distress, anxiety, loneliness, access to transport, wellbeing, hunger, thirst Conflicting evidence: dental status, swallowing, cognitive function, depression, residential status, medication intake and/or polypharmacy, constipation, periodontal disease
van der Pols-Vijlbrief [34], 2014 USA, Canada, Netherlands, Sweden, Cuba, France, Japan, Brazil, UK, Israel, Russia, 28 studies *N* = 49–12,883, mean > 65y	21.3–56.5%, CD, N/A	PEM (WL over time/ low nutritional intake/ low BW/ poor appetite)	Demographic: sex, age, education Food Intake: reduced appetite, edentulousness, chewing difficulties Lifestyle: PA, alcohol use, smoking Social: few friends, living situation, loneliness, death of spouse Physical functioning: ADLs Psychological: depression, cognitive decline, dementia, anxiety Disease-related: hospitalisation, SR health status, polypharmacy, chronic diseases, cancer	N/A	MV analyses	Association: poor appetite Moderate evidence for an association: edentulousness, hospitalization, SR health moderate evidence for no association: older age, low education, depression, chronic diseases Strong evidence for no association: few friends, living alone, loneliness, death of spouse No association: chewing difficulties, alcohol consumption, anxiety, number of diseases, heart failure, use of anti-inflammatories Inconclusive: sex, low PA, smoking, ADL dependency, cognitive decline, dementia, polypharmacy
**Meta-analysis**						
Streicher [33], 2018, 6 studies: Germany (30, Ireland (1), Netherlands (1), New Zealand (1), *N* = 209–1841, 71.7 (5.0)- 84.6 (0.5)	36.6–50.5%, CD, N/A	MN (BMI < 20 kg/m^2^ or WL > 10% over FU)	Demographic: age, sex, marital status, education Social: living alone, social support Lifestyle: PA, smoking status, alcohol consumption Disease-related: comorbidities (> 2), hospitalisation (6 m/1 y before baseline and 6 m/1 y before FU), pain, SR health, polypharmacy (> 5 drugs) Psychological: cognitive impairment (MMSE < 23, TICS-m < 31), depression (GDS > 6, CES-D > 16, HADS > 8) Physical functioning: difficulty walking, difficulty climbing stairs, HGS, falls (y before baseline and 1 y/2 y before FU) Food intake: appetite	1–3 y	LR analyses (UV and MV), random-effects meta-analyses	increasing age [1.05 (1.03–1.07) *p* < 0.05]; unmarried, separated, or divorced status [1.54 (1.14–2.08) *p* < 0.05]; difficulty walking 100 m [1.41 (1.06–1.89) *p* < 0.05]; difficulty climbing stairs [1.45 (1.14–1.85) *p* < 0.05]; hospitalisation before baseline [1.49 (1.25–1.76) *p* < 0.05]; hospitalisation during FU [2.02 (1.41–2.88) *p* < 0.05]

*A* accelerated, *AACI* Charlson’s Age Adjusted Co-Morbidity Index, *ADL* activities of daily living, *AGGIR* Autonomy, Gerontology and Group Resources Scale, *ANOVA* analysis of variance, *ANSI* Australian nutritional screening initiative, *BI* Barthel Index, *BMI* body mass index, *BOHSE* Brief Oral Health State Examination, *BP* blood pressure, *BW* body weight, *CCI* Charlson Comorbidity Index, *CD* community dwelling, *CDS* chronic disease score, *CESD* center for epidemiologic studies depression scale, *CHD* coronary heart disease, *CI* confidence interval, *CIDI* Composite International Diagnostic Interview, *CNAQ* Council on Nutrition Appetite Questionnaire, *CPS* Cognitive performance scale, *CRP* C-reactive protein, *CVD* cardiovascular disease, *DM* diabetes mellitus, *EAT-10* Eating Assessment Tool-10, *EI* energy intake, *ENS* elderly nutrition screening, *EPI* Eysenck Personality Inventory, *EQ-5D* euro quality of life- 5 dimension, *ESDS* Enforced Social Dependency Scale, *FFMI* fat free mass index, *FTSTS* Five-times-sit-to-stand test, *FU* follow up, *GDS* geriatric depression scale, *GFI* Groningen Frailty Index, *GOHAI* Geriatric Oral Health Assessment Index, *GP* general practitioner, *HADS* Hospital Anxiety and Depression Scale, *HDDS* Household Dietary Diversity Score, *HEI* Healthy Eating Index, *HFIAS* Household Food Insecurity Access Scale, *HGS* handgrip strength, *HR* hazards regression, *HTAQ* Health and Taste Attitudes Questionnaire, *HUI* Health Utility Index, *IADL* instrumental activities of daily living, *invol* involuntary, *IM-E-SA* INTERMED questionnaire for the Elderly Self-Assessment, *IQCODE* Informant Questionnaire on Cognitive Decline in the Elderly, *IQR* interquartile range, *LAPAQ* Longitudinal Aging Study Amsterdam (LASA)-Physical Activity Questionnaire, *lb* pound, *LR* logistic regression, *LSNS-6* Lubben social network scale-6, *m* months, *MAG* Malnutrition Advisory Group, *MCI* mild cognitive impairment, *MCS* mental component score, *MEDAS* Mediterranean Diet Adherence Screen, *MI* myocardial infraction, *min* minute, *MMSE* mini mental state examination, *MN* malnutrition, *MNA* mini nutritional assessment, *MNA-SF* mini nutritional assessment- short form, *MoCA* Montreal Cognitive Assessment, *MOS* Medical Outcomes Study, *MOW* meals on wheels, *MUAC* mid-upper arm circumference, *MUST* malnutrition universal screening tool, *MV* multivariate, *NEADL* Nottingham Extended Activities of Daily Living, *NHP* Nottingham health profile, *NIDS* National Irish Deprivation Score, *NRS-2002* Nutritional Risk Screening, *NUFFE* Nutritional Form For the Elderly, *NUFFE-NO* Norwegian version of the Nutritional Form For the Elderly, *OARS* Older Americans Resources and Services, *OHQ* oral health questionnaire, *OR* odds ratio, *OSLO 3-SSS* Oslo 3 item social support scale, *PA* physical activity, *PASE* Physical Activity Scale for the Elderly, *PCS* physical component score, *PEM* protein energy malnutrition, *PGC MAI* Philadelphia Geriatric Centre Multilevel Assessment Instrument, *QOL* quality of life, *RR* risk ratio, *S* successful, *SASE* Self-care Ability Scale for Elderly, *SC* senior centre, *SCL-10* symptoms check list- 10, *SCREEN* Seniors in the community: Risk Evaluation for eating and Nutrition, *SD* standard deviation, *SEM* structural equation modelling, *SF-12* short form survey-12, *SF-36* short form survey-36, *SHPS* Subjective Health Perceptions Scale, *SNAQ*^*app*^ Simplified Nutritional Appetite Questionnaire, *SNAQ*^*65+*^ Short Nutritional Assessment Questionnaire for over 65 s, *SOC* Sense of coherence scale, *SOF* Study of osteoporotic fractures, *SPMSQ* Short-Portable Mini-Mental Status Questionnaire, *SR* self rated, *SSQSF* Social Support Questionnaire- Short Form, *STAI Y-B* State-Trait Anxiety Inventory form Y, *TICS-m* modified Telephone Interview for Cognitive Status, *TUG* Timed Up and Go, *U* usual, *U-A* usual to accelerated, *U-S* usual to successful, *UCLA* University of California at Los Angles, *UV* univariate, *VA* Veterans Administrative, *VAS* visual analogue scale, *WC* waist circumference, *WHOQOL* world health organisation quality of life scale, *WL* weight loss, *y* years, *3MS* Modified Mini-Mental State

^a^ answering ‘yes’ to either an ADL/IADL was categorised as ‘need’; ^b^ assessed using PGC-MAI: measures cognition, physical health, mobility, ADLs, time use, personal adjustment, social interaction and environmental domains; ^c^ dependent for activities of daily living (grocery shopping, transportation, cooking, or self-care); *Key results are only presented for multivariate analyses

### Categorisation of studies according to rate of ageing

Nine studies were classified as ageing at a usual rate [35, 36, 39, 49, 63, 66, 70, 84, 99]. Three studies were classified as ageing successfully and five studies were categorised as ageing at an accelerated rate. Six studies were placed between the successful and usual ageing groups [34, 41, 42, 45, 68, 100] and five studies were placed between the usual and accelerated ageing categories [38, 44, 53, 77, 93]. In order to include as many studies as possible in our results, studies classed within the usual to successful ageing category were collapsed into the successful ageing category [21, 85, 87] whilst studies within the usual to accelerated category were collapsed into the accelerated ageing category [40, 46, 94–96] (Fig. 2). Forty studies remained uncategorised so were omitted from the synthesis of studies by ageing rate; however, the details of each of these studies are described in Table 2. Primary reasons for not categorising studies included lack of information on presence of chronic diseases, polypharmacy, functionality, frailty or use of social or medical services not being provided or that the study included multiple cohorts (details of all studies included in this review are within Table 2).

### Factors associated with, and determinants of, malnutrition

Factors in the demographic and disease-related domains were most-commonly examined (63 and 54 studies respectively), followed by the social (50 studies), psychological and physical functioning domains (46 studies each) (Table 2). Factors under the food intake and lifestyle domains were the least well studied (32 and 20 studies respectively). The factors most-commonly reported to be associated with malnutrition were within the demographic (41 studies), disease-related (34 studies), physical functioning (30 studies) and psychological (30 studies) domains. Domains less commonly reported as associated with malnutrition were the social (27 studies), food intake (23 studies) and lifestyle (7 studies). The evidence for individual factors within each domain is critically considered.

The frequency of factors reported as associated with malnutrition according to the rate of ageing category is presented in Table 3. In this review, demographic factors such as being female (successful, *N* = 2; usual, *N* = 1; accelerated, *N* = 1) and increasing age (successful, *N* = 2; usual, *N* = 3; accelerated, *N* = 1) were commonly reported as associated with malnutrition/malnutrition risk across all ageing rate categories. Other demographic (unmarried status (*N* = 4) [42, 45, 85, 100] and a low education level (*N* = 2) [34, 68]) and physical functioning factors were more commonly reported within the successful ageing category compared to the other ageing rate categories. Factors within the food intake and disease-related domains were most-commonly reported in older adults who are ageing at an accelerated rate.

**Table 3:** Factors associated with malnutrition in community-dwelling older adults stratified by ageing rate

Domain	Successful (***N*** = 9)	Usual (***N*** = 9)	Accelerated (***N*** = 10)
Demographic	**Female (2), marital status (4), age (2),** BMI (1), **education (2),** ethnicity (1)	**Age (3),** BMI (3), WL (1), measures of fat mass (1), **female (1), marital status (2),** rural location (1)	**Female (1), age (1),** unintentional WL (1), rural location (1), ethnicity (1), **marital status (1)**
Lifestyle	PA (1)	Alcohol (1)	Smoking (1), PA (1)
Food Intake	Appetite (1), **food insecurity (1)**	Appetite (1), oral health (1), illness which affects food intake (1), normal swallow (RR) (1), **choosing food that’s easy to chew (1)**	Appetite (2), < 3 snacks per day (1), oral health (1), **eating difficulty (1), eating dependency (1)**
Social	**Social support** (2), **living situation** (1), income (1)	**Living alone** (2), living with another adult (1), income (3), **low social support (2), social isolation (2),** requiring assistance to travel (1), availability of reliable transport (1), religious attendance (1), fear of attack (1), fear discrimination (1)	Income (1), **MOW (2), increasing use of MOW (1), help making meals (1)**
Physical Functioning	**difficulty walking/climbing stairs (2), falls (1),** orthopaedic disability (1)	Healthy physical performance (1), IADL (1), moderate/severe disability (1)	**Unable to go outside (1), ADL (2),** functional status (1), **falls (1),** IADL (RR) (1)
Psychological	**Cognitive impairment (1)**, **depression (1),** mental health (1)	**Dementia (1), depression (2)**	**Depression (2), dementia (1),** mental overload (1), cognitive impairment-men (1)
Disease-related	SR health (2), **hospitalisation (1),** low medication use (RR vs none) (1), multi-morbidity (2), QoL (1), physical health (1)	Polypharmacy (3), SR health (2), institutionalisation (1), not regularly attending GP (RR) (1), health problems (1), general health status (1)	Intestinal problems (1), multi-morbidity (1), **osteoporosis (1),** nausea (1), **cancer (1),** health related QoL (1), poor SR health (1), **hospitalisation (2), > 2 emergency hospitalisation (1), hospital stay > 4 weeks (1)**

*ADL* activities of daily living, *MOW* meals on wheels, *PA* physical activity, *QoL* quality of life, *RR* reduced risk, *SR* self-rated, *WL* weight loss

Bold text indicates factors which are more frequently reported as the rate of ageing increases

This review found that factors reported to be associated with malnutrition from the food intake domain increased in frequency and severity across the three ageing categories (successful, usual, accelerated). Food insecurity was reported as a risk factor in the successfully ageing category [42], choosing foods that were easy to chew was a risk factor in the usual ageing category [39], whilst difficulties eating and eating dependency were associated with malnutrition risk in the accelerated ageing category [77]. Having a poor or reduced appetite is reported as being associated with malnutrition or malnutrition risk across all categories of ageing rate [39, 44, 77, 87].

Within this review, lifestyle factors were rarely reported as being associated with malnutrition or malnutrition risk in any of the ageing categories. Lack of physical activity was reported once in both the successfully [68] and accelerated [46] ageing categories. Alcohol use was reported as being associated with a lower risk of malnutrition once within the usual ageing category [49]. Smoking was reported to be associated with malnutrition in one study from the accelerated ageing category [46].

Cognitive impairment, a factor within the psychological domain was reported as being associated with malnutrition by one study in the successful ageing category [85], whilst dementia was reported as associated with malnutrition risk in both the usual (*N* = 1) [36] and accelerated (*N* = 2) [53, 94] ageing categories. Depressive symptoms were reported in the successful ageing (*N* = 2) [34, 45], usual ageing (*N* = 3) [35, 36, 49] and accelerated ageing (*N* = 2) [38, 53] categories.

Indicators of declining mobility (difficulty walking 100 m and difficulty climbing a flight of stairs) were reported in the successful ageing category only (*N* = 2) [85, 87]. Factors indicative of physical dependency (being unable to go outside) were reported in one study from the accelerated ageing category [46]. Falls were reported to be associated with malnutrition or malnutrition risk in the successful ageing (*N* = 1) [85] and accelerated ageing (*N* = 1) [96] categories.

Living with others was associated with reduced risk of developing malnutrition in the successful ageing category (*N* = 1) [45], whilst living alone was associated with increased risk of malnutrition risk in the usual ageing category (*N* = 2) [35, 39]. Social support was reported to be associated with malnutrition or malnutrition risk in both the successful (*N* = 2) [85, 100] and usual (*N* = 2) [35, 99] ageing categories.

This review found factors from the disease-related domain were commonly reported across all ageing rate categories but increased in severity as the ageing rate progressed into the accelerated ageing category. Recent hospitalisation was reported in the successful (*N* = 1) [85] and accelerated (*N* = 2) [94, 96] ageing categories. Factors such as multi-morbidity were more commonly reported in the successful ageing category (*N* = 2) (*N* = 0, usual ageing category, *N* = 1, accelerated ageing category) whilst individual diseases such as cancer and osteoporosis (*N* = 1) [46] and extended hospital stays (*N* = 1) [96] were reported in the accelerated ageing category.

## Discussion

This review provides a summary of the factors associated with malnutrition and malnutrition risk reported in community-dwelling older adults with an emphasis on differences identified according to rate of ageing [15]. This novel approach has found that as the rate of ageing accelerates, an increasing number of factors are reported within the food intake, social and disease-related domains; and these factors increase in severity in the accelerated ageing category. Within the usual and accelerated ageing categories, dementia is reported to be associated whilst cognitive impairment appears in the successful ageing category. Indicators of declining mobility and function are associated with malnutrition and these indicators increase in severity across the ageing categories. Within the successful ageing category, demographic factors such as low education level and unmarried status appear to be most important. Factors such as hospitalisation and falls appear to be relevant regardless of rate of ageing.

The findings presented in this paper contribute to our understanding of the factors associated with, and determinants of, malnutrition in older adults and may explain differences in factors associated with, and determinants of, malnutrition reported in previously published studies. Standardised criteria for the diagnosis of malnutrition were only published as recently as 2019 [26, 27]. The majority of studies included in this review were published prior to this date; thus, many differing definitions of malnutrition were used. The lack of consistency between studies makes comparisons difficult; however, implementation of these 2019 criteria in future studies should help to reduce the heterogeneity.

### Factors associated with, and determinants of, malnutrition

#### Demographic domain

Numerous cross-sectional studies included in this review reported no association between marital status and malnutrition [34, 37, 46, 47, 50, 51, 62, 70, 88, 93]. Conversely, other studies, including a recent meta-analysis of longitudinal studies, did report a relationship, whereby not being married was associated with an increased risk of developing malnutrition [33, 42, 45, 56, 73, 76, 95, 99]. This may be attributed to the fact that being married is linked to better health behaviours across life, with this effect being more pronounced in men [52]. In this review, unmarried status was frequently reported to be associated with malnutrition or malnutrition risk in the successful ageing category. Most of the evidence in this review suggested that level of education is not associated with malnutrition [32, 33, 35, 36, 38, 42, 45, 46, 49, 50, 54, 55, 61, 62, 66, 67, 70, 71, 73, 76, 85, 87, 88, 93, 99–102]. However, when stratified by rate of ageing, a low level of education appeared to be more commonly reported as being associated with malnutrition within the successful ageing category. These demographic factors could be playing a key role in the development of malnutrition within the successful ageing group as older adults in this category are not burdened with chronic diseases, mental or physical functional limitations to the same extent as older adults in the other ageing rate categories.

Age and female sex are reported to be associated with malnutrition and malnutrition risk across all ageing rate categories. It has been reported that females have a 45% higher chance of developing malnutrition compared to their male counterparts [72]. This could be due to a multitude of factors including the fact that globally women have longer life expectancies than men [72, 82]. Women are also more likely to experience adverse social and economic circumstances in old age [72, 103–105], which are themselves independently associated with increased risk of malnutrition. Within the included studies, many reported an independent association between increasing age and deteriorating nutritional status [34, 42, 43, 53, 57, 62, 63, 76, 79, 81, 83, 88, 106, 107]; conversely, a systematic review concluded there was moderate strength evidence to suggest that older age and malnutrition are not associated [32]. Furthermore, a second systematic review concluded that it is likely that frailty is driving the association seen between malnutrition and advancing age [89]. Factors within the demographic domain are frequently reported to be associated with malnutrition; however, consideration should be given as to whether these are true determinants of the condition or whether the associations seen are false positives due to frequency of assessment.

#### Food intake domain

Factors affecting food intake, such as the amount of food eaten or the ability to eat/feed oneself, appear to be particularly associated with malnutrition within the accelerated ageing category, compared to the other categories. This may be in line with the fact that this group comprises a sicker, and more diseased population group. The escalation in the severity of these factors across the ageing categories (from food insecurity to factors affecting food choice to having difficulty or being unable to self-feed), highlights that as older adults deteriorate in health and function, they become more vulnerable to developing malnutrition.

In this review, a reduced/poor appetite appears to be associated with malnutrition across all ageing rate categories. Reduced appetite can be a consequence of many factors known or suggested to be associated with, or determinants of, malnutrition, including depression, cognitive decline, chewing or swallowing difficulties and sensory changes [90, 108, 109]. Two systematic reviews included in this review reported that reduced appetite is associated with malnutrition with one of these reviews reporting that poor appetite was the only factor that had strong evidence to support an association with malnutrition [32, 110]. Conversely however, a meta-analysis of longitudinal studies reported no association with incident malnutrition [73]. These differences may be related to the fact most studies included in the systematic reviews and categorised by rate of ageing in this review were cross-sectional in design, whilst the meta-analysis only included longitudinal studies. In addition, variances in the way the question on appetite was asked between studies may have contributed to these differences.

Evidence surrounding the association between dental status and presence of chewing problems and malnutrition is conflicting [39, 44, 55, 61, 63, 79, 93, 110, 111]. Having no/few teeth and difficulties chewing can be detrimental to diet quality and lead to malnutrition as nutrient-dense foods (e.g., meat, fruit and vegetables) may be avoided in favour of softer, higher calorie but less nutrient-dense foods which may be easier to eat [98]. However, difficulties chewing or swallowing may also be a consequence of malnutrition as a decline in physical function is a known outcome of malnutrition which may explain the conflicting findings found amongst the cross-sectional studies included in this review [41, 44, 63, 97].

#### Lifestyle domain

Lifestyle factors were seldom reported across all categories of ageing rate; thus, the evidence surrounding lifestyle factors, such as alcohol consumption, smoking and low physical activity and malnutrition is weak. Few associations have been reported for physical activity as a protective factor [51, 56, 68] and smoking as increasing risk [33, 46] of malnutrition in cross-sectional studies. One cross-sectional study reported alcohol intake as protective against malnutrition [49]. This study was conducted in The Netherlands which is one of the lowest alcohol-consuming countries in Europe; therefore, this finding may not be applicable in countries with higher consumption rates [112]. All other included studies, including a meta-analysis and two systematic reviews, failed to report associations between alcohol consumption and malnutrition [32, 37, 46–49, 70, 73, 85, 87, 96, 110, 111, 113]. As reported in a previous systematic review [110], our review reinforces the conclusion that factors within the lifestyle domain do not appear to be determinants of malnutrition in older adults.

#### Social domain

Factors within the social domain, predominantly factors related to social support, were apparent within the successful and usual ageing categories, where social factors related to use of services were more prevalent amongst the accelerated ageing category, likely reflecting increased dependency among this group and subsequently, a higher need for these services. This finding is supported by two longitudinal studies which reported that meals-on-wheels use, which may be linked to reduced social (and physical) functioning, was associated with increased risk of malnutrition [40, 94]. Amongst fit, community-dwelling older adults, those with the highest levels of social vulnerability (defined using the social vulnerability index) have been reported to be more than twice as likely to die compared to their counterparts who had the lowest levels of social vulnerability [58]. In contrast, a meta-analysis has reported that living alone or receiving social support do not predict incident malnutrition [73]. These differences may be related to study design as our review is predominantly comprised of cross-sectional studies.

#### Physical functioning domain

Evidence surrounding a relationship between inability or difficulty completing activities of daily living (ADLs) and malnutrition is conflicting [34, 36, 43, 45, 46, 49, 50, 63, 71, 83, 88, 92–95, 97, 111]. A systematic review has stated there was inconclusive evidence to identify whether there was an association with malnutrition [32]. This conflicts with other studies which suggest that declining health and/or functionality can make cooking, personal transport and grocery shopping difficult; therefore, negatively affecting nutritional status [114]. Further work is required to fully understand this.

Low handgrip strength (HGS) did not appear to be associated with malnutrition across any of the rate of ageing categories. Furthermore, HGS, was reported to have no association with incident malnutrition following a meta-analysis of longitudinal cohorts [73]. Although this may seem surprising as HGS is often used as a marker for functionality and/or frailty, it may be explained by the fact that declines in physical function are a known outcome of malnutrition and, therefore, low HGS is likely a consequence as opposed to a determinant of the condition. As such, low HGS may be a useful indicator of those who are severely malnourished as opposed to those exhibiting early signs or risk of developing malnutrition.

Falls among older adults, can be an indicator of declining cognition or onset of frailty [91, 115] and can result in fractures and hospitalisation, known risk factors for nutritional decline [116, 117]. Increased risk of falling appears to be associated with malnutrition in both the successful ageing and accelerated ageing groups, suggesting a bidirectional relationship between falling and malnutrition, whereby it could be a determinant of malnutrition for an older person ageing at a successful rate, initiating a rapid deterioration in health. Equally, it could be a consequence of malnutrition in an older person ageing at an accelerated rate. Adding weight to this hypothesis, a recent meta-analysis of six longitudinal studies reported no association between falls and incident malnutrition. However, this study reported that difficulty walking 100 m and difficulty climbing a flight of stairs (indicators of mobility) were determinants of incident malnutrition [73]. Indicators of declining mobility associated with malnutrition appear to increase in severity across ageing rate categories. Difficulties walking 100 m or climbing a flight of stairs appeared as associated factors in the successful ageing group whilst being unable to go outside is an associated factor in the accelerated ageing category.

#### Psychological domain

The prevalence of malnutrition is significantly higher among people with dementia; however, this is more likely to impact on the determinants of malnutrition in long-term care settings where dependency is higher compared to the community setting [118, 119]. Difficulties assessing whether cognitive decline is a determinant of malnutrition are compounded by the under-representation of this cohort of older adults within studies. Cognitive decline appears to be associated with malnutrition in one study in the successful ageing category, while dementia is associated with malnutrition within the usual ageing and accelerated ageing categories. It is likely that this is signifying the progressive decline in health as older adults move from ageing at a successful rate into the other less successful ageing categories.

#### Disease-related domain

Disease-related factors appear across all ageing categories; however, specific diseases such as cancer and osteoporosis only appear within the accelerated ageing category. Malnutrition is common among older adults with cancer, with the prevalence ranging from 30 to 85% depending on the cancer type [120]. Recent hospitalisation is the factor most likely to impact negatively on an older person’s nutritional status within the disease-related domain [32, 73, 110]. Hospitalisation appears as an important factor within the successful and accelerated ageing categories in this review. However, prolonged hospital stay (> 4 weeks) only appears as a factor within the accelerated ageing category. Similar to falls, hospitalisation is likely to have a bidirectional relationship with malnutrition, being a determinant of the condition for those ageing at a successful rate and a consequence for those within the accelerated ageing category.

Numerous studies have reported associations between poor self-rated (SR) health and malnutrition [32, 37, 42, 47, 81, 84, 88, 94, 106, 110, 121, 122] with SR health being a prevalent factor across all ageing rate categories in this review. This contrasts with a recent meta-analysis (of longitudinal studies) that reported no association between SR health and incident malnutrition [73]. Contradictory results have been reported surrounding the relationship between polypharmacy and the number of chronic diseases with malnutrition and malnutrition risk. Two systematic reviews have concluded that the evidence for polypharmacy as a factor associated with malnutrition was inconclusive and that there was moderate evidence to support no association with number of comorbidities [32, 110]. The conflicting results reported for these factors is likely due to the differing numbers of medications/diseases being used to define polypharmacy/multimorbidity between studies.

## Strengths and limitations

This review used a novel approach of categorising community-dwelling older adults according to their rate of ageing (successful, usual or accelerated) and assessed whether differences occurred in the factors associated with malnutrition for each category. To the best of our knowledge, no other study has taken this approach previously. This approach may contribute to reducing the heterogeneity in factors reported to be associated with malnutrition among older adults in the community setting.

There are a number of limitations associated with the published literature on the determinants of malnutrition in older adults. Whilst 68 studies were initially identified as relevant for inclusion in this review, 40 could not be categorised according to rate of ageing due to the lack of detailed information on the characteristics of the study population provided within the published manuscripts. These studies were, therefore, omitted from the synthesis of factors associated with, and determinants of, malnutrition by rate of ageing. Had these manuscripts contained sufficient information to permit categorisation by rate of ageing, our results would have been strengthened or potentially different. Where possible, the current review sub-categorised the study populations from the included studies into successful, usual or accelerated ageing. It is likely that there was heterogeneity between the participants included in individual studies; however, group means were used to categorise the populations from individual papers as a whole into rate of ageing categories. Furthermore, there was variation in the parameters used in different studies, for example, number of medications to define polypharmacy and method of measuring functional independence.

The majority of studies included in this review used convenience samples, often with small sample sizes. This limits the use of the results as they cannot be extrapolated to represent the general population of community-dwelling older adults. A number of studies only investigated factors from one or two domains, thus, failing to acknowledge the multifactorial aetiology of malnutrition. Furthermore, a factor or determinant could not be identified as associated with malnutrition if it had not been included in the original study. The majority of current published literature is cross-sectional in design. Studies of longitudinal design are superior to definitively determine the factors which predict malnutrition as cross-sectional studies cannot distinguish between the causes and consequences of malnutrition. This review included only studies published in the English language and from the year 2000 onwards. The timeframe chosen was to ensure a more standardised approach to the identification of malnutrition and malnutrition risk and allowed for the identification of potential factors and determinants of malnutrition relevant to the health of older adults in the past 20 years. Nonetheless, these factors may have introduced selection bias into our results.

## Conclusions

Numerous changes occur with ageing, increasing the vulnerability of older adults to developing malnutrition. Older adults are a heterogeneous group; thus, assessment of individuals’ rate of ageing could aid in identifying specific determinants for different cohorts of community-dwelling older adults. In the future, categorising community-dwelling older adults according to their rate of ageing could also be incorporated into malnutrition screening methods; this would allow for a more personalised approach to identifying malnutrition in older adults as different domains and different individual factors appear to be important depending on the ageing category. Further longitudinal studies and meta-analyses, segregating elderly by ageing rate, are warranted to clearly distinguish which factors are true determinants of malnutrition and not simply the consequences of the condition.

## Data Availability

Not applicable.

## Abbreviations

ADL: Activities of daily living
BMI: Body mass index
ESPEN: European society of Clinical nutrition and metabolism
FFM: Fat free mass
GP: General practitioner
HCP: Healthcare professional
HGS: Handgrip strength
SR: Self-rated
